# CHH Methylation Islands: A Nonconserved Feature of Grass Genomes That Is Positively Associated with Transposable Elements but Negatively Associated with Gene-Body Methylation

**DOI:** 10.1093/gbe/evab144

**Published:** 2021-06-19

**Authors:** Galen T Martin, Danelle K Seymour, Brandon S Gaut

**Affiliations:** 1 Department of Ecology and Evolutionary Biology, University of California, Irvine, California, USA; 2 Department of Botany and Plant Sciences, University of California, Riverside, California, USA

**Keywords:** DNA methylation, Poaceae, mCHH islands, comparative analysis, epigenetics, transposable elements

## Abstract

Methylated CHH (mCHH) islands are peaks of CHH methylation that occur primarily upstream to genes. These regions are actively targeted by the methylation machinery, occur at boundaries between heterochromatin and euchromatin, and tend to be near highly expressed genes. Here we took an evolutionary perspective by studying upstream mCHH islands across a sample of eight grass species. Using a statistical approach to define mCHH islands as regions that differ from genome-wide background CHH methylation levels, we demonstrated that mCHH islands are common and associate with 39% of genes, on average. We hypothesized that islands should be more frequent in genomes of large size, because they have more heterochromatin and hence more need for defined boundaries. We found, however, that smaller genomes tended to have a higher proportion of genes associated with 5′ mCHH islands. Consistent with previous work suggesting that islands reflect the silencing of the edge of transposable elements (TEs), genes with nearby TEs were more likely to have mCHH islands. However, the presence of mCHH islands was not a function solely of TEs, both because the underlying sequences of islands were often not homologous to TEs and because genic properties also predicted the presence of 5′ mCHH islands. These genic properties included length and gene-body methylation (gbM); in fact, in three of eight species, the absence of gbM was a stronger predictor of a 5′ mCHH island than TE proximity. In contrast, gene expression level was a positive but weak predictor of the presence of an island. Finally, we assessed whether mCHH islands were evolutionarily conserved by focusing on a set of 2,720 orthologs across the eight species. They were generally not conserved across evolutionary time. Overall, our data establish additional genic properties that are associated with mCHH islands and suggest that they are not just a consequence of the TE silencing machinery.

Significance

Plants methylate cytosines within DNA sequences. Methylation serves different functional purposes depending on the pattern and location in the genome. Recent work has documented that islands of CHH methylation sometimes occur near plant genes and that the presence of an island correlates positively with gene expression. However, there is little knowledge about what causes these islands, their potential function, and their evolutionary dynamics. We investigate this epigenetic puzzle by characterizing upstream methylation islands in eight species of grasses. We find that body-methylated genes are less apt to have mCHH islands, that long genes and genes near TEs tend to have islands more often, that the association of islands with gene expression is generally weak, and that islands are rarely evolutionarily conserved between species.

## Introduction

 Epigenetic marks—such as DNA methylation, histone modifications, and nucleosome positioning—affect the function and evolution of plant genomes (Diez et al. 2014; Vidalis et al. 2016). Perhaps the best characterized epigenetic effect is transposable element (TE) silencing. Epigenetic silencing within TEs is achieved through a complex series of biochemical reactions that usually result in the methylation of cytosines in three contexts: CG, CHG, and CHH (where H = C, T, or A). Methylation in all three contexts is associated with transcriptional silencing and a heterochromatic state, which effectively renders a TE unable to propagate (Slotkin and Martienssen 2007; Fultz et al. 2015). This silencing has evolutionary effects both because it alters the potential trajectory of genome content, which is dominated by TEs in large plant genomes (Lee and Kim 2014), and because TE methylation affects the expression of nearby genes (Lippman et al. 2004; Choi and Lee 2020).

The processes of DNA methylation and maintenance vary by cytosine context. In *Arabidopsis thaliana*, CG methylation is deposited and then maintained across generations by the DNA methyltransferase MET1 (see Law and Jacobsen 2010 for a review). Once it is established, CHG methylation is maintained by a separate methyltransferase (CMT3). In contrast to CG and CHG methylation, CHH methylation is not maintained but must be deposited de novo every generation. This deposition is achieved by one of two pathways. One is RNA-directed DNA methylation (RdDM), which uses homology of small-interfering RNAs (siRNAs) to guide methyltransferase machinery to complementary DNA sequences (Law and Jacobsen 2010). At siRNA target sites, the methyltransferase enzyme deposits methylation in all three contexts (CG, CHG, and CHH), particularly at the edges of targeted TEs (Gent et al. 2013; Zemach et al. 2013). The second pathway includes the plant-specific methylases *CHROMOMETHYLASE 2* (*CMT2*) and *CHROMOMETHYLASE 3* (*CMT3*) (Gouil and Balcombe 2016), which methylate CHH and CHG cytosines in deep heterochromatin (Bewick et al. 2017). Unlike RdDM, *CMT2* tends to methylate TEs across their full length (Zemach et al. 2013), but the regions methylated by RdDM and *CMT2* do frequently overlap in *A.thaliana* (Zemach et al. 2013).

These pathways contribute to the epigenetic features known as methylated CHH (mCHH) islands. mCHH islands are short regions of elevated methylation typically found upstream and downstream from genes. mCHH islands were first identified in rice, where they were associated with miniature inverted-repeat transposable elements (Zemach et al. 2010b), a group of terminal inverted repeat (TIR) DNA elements that often insert near genes. mCHH islands have also been characterized in maize (*Zea mays* ssp. *mays*); they were located near ∼50% of genes and tend to be nearby genes with high expression levels (Gent et al. 2013). The maize analyses suggest that mCHH islands do not represent typical TE methylation, because maize TEs within 1 kb of genes are more heavily CHH methylated than other TEs and are more heavily methylated on the side of the TE closest to the gene that contained the mCHH island.

Thus far, the function of these mCHH islands is unclear. Given that they occur along boundaries between euchromatin and heterochromatin and also that mCHH island-associated genes tend to be more highly expressed than other genes, Gent et al. (2013) proposed that they partition the genome between different chromatin states, either by preventing the spread of epigenetic modifications into genes or, vice versa, by preventing the spread of euchromatin into TEs, thereby potentially reactivating them. Li et al. (2015) explored this potential function using *mop1* maize mutants, which lack mCHH islands. They confirmed that the loss of RdDM leads to an increase of transcribed RNA from some TEs (between 29 and 179, depending on the tissue examined), suggesting that mCHH islands contribute to TE silencing. Similarly, others have found that the loss of near-gene RdDM in *mop1* mutants can lead to unstable TE silencing that may be more susceptible to spontaneous reactivation during heat stress (Guo et al. 2021). Nonetheless, these observations do not fully explain why mCHH islands are concentrated near expressed genes. One potential explanation is that mCHH islands are a result, rather than a cause, of gene expression; this explanation is consistent with the observation *mop1* mutants do not display widespread downregulation of mCHH-deficient genes (Li et al. 2015). There is, however, some evidence for a causal relationship between mCHH islands and gene expression, because recent work has shown that two *A. thaliana* gene products (SUVH1 and SUVH3) form a complex that binds CHH methylated sequences and enhances transcription (Harris et al. 2019). Raju et al. (2019) suggest that this mechanism implicates mCHH islands in protecting and promoting the expression of genes nearby TEs.

Until recently, it has been unclear whether mCHH islands are an idiosyncrasy of rice and maize or instead a general feature of plant epigenomes. To address this question, Niederhuth et al. (2016) surveyed genome-wide methylation patterns across a panel of 34 angiosperms. They defined mCHH islands as 100 bp windows within 2 kb of genes that were methylated in at least 25% of reads mapped to cytosines in the CHH context. This definition was based on previous work (Li et al. 2015), but it did not account for the widely varying background levels of CHH methylation found across species. Their survey also predominantly contained genomes of relatively small size. Their survey did include the TE-rich ∼2.3 Gb maize genome, but the remaining species had genomes of <1.25 Gb in size, which is much smaller than the angiosperm average of 5.7 Gb (Dodsworth et al. 2015). This size distribution makes it difficult to assess whether mCHH islands correlate with genome size, as do other features of plant epigenomes (e.g., Alonso et al. 2015; Niederhuth et al. 2016; Takuno et al. 2016). Nonetheless, the Niederhuth et al. (2016) survey was remarkably informative about many aspects of DNA methylation variation among angiosperms, including mCHH islands. It reported, for example, that species vary markedly in the percentage of genes associated with upstream mCHH islands, from <1% in *Vitis vinifera* to ∼74% in *Beta vulgaris*. They also found that several species did not demonstrate an obvious association between mCHH islands and gene expression, making the relationship unclear.

Here we study mCHH islands in members of the grass family (Poaceae). We have chosen to focus on grasses for several reasons, including that they are economically important, that their intermediate evolutionary age makes them a useful comparative system, and that they encompass extensive variation in diploid genome size. They are also an interesting system from the perspective of CHH methylation, because all of the grass species surveyed thus far have low background levels of CHH methylation compared with other angiosperms (Bewick et al. 2017). This is a useful property for studying mCHH islands, because they can be easily detected as exceptions to the background pattern of low CHH methylation.

To study mCHH islands, we focus on a set of eight grass taxa that span the breadth of the family, that vary widely in genome size, and that have available data—that is, whole-genome bisulfite sequencing (WGBS) data and RNAseq data (Seymour and Gaut 2020). Importantly, 2,720 1-to-1 orthologs have been identified among these same taxa, so that we can assess the evolutionary conservation of mCHH islands across species for specific genes. Given these data, we identify mCHH islands using methods that recognize that genome-wide mCHH levels vary across species. We then address four questions: First, what is the genome-wide pattern of mCHH islands across species? Is there, for example, a correlation with genome size for mCHH islands, as for other features of DNA methylation? Second, are islands located near genes that have nearby TEs, reinforcing the notion that mCHH islands are associated with TEs? Third, is there a relationship between mCHH islands and gene expression? That is, does gene expression predict the presence of a nearby island, or do other genic features better predict an island’s presence? Finally, we take advantage of orthologous genes to investigate whether mCHH islands are evolutionarily conserved across species. Once established, is an island conserved, or is it an evolutionarily short-lived feature of the epigenomic landscape?

## Materials and Methods

### Data and Methylation Calls

These analyses used RNAseq and BSseq data from eight grass species. The *Hordeumvulgare*, *Triticumurartu*, *Setariaitalica*, and *Sorghum bicolor* data were retrieved from the NCBI Short Read Archive under accession PRJNA340292, all of which were generated from leaf tissue in 6-week-old plants. Data from *Brachypodiumdistachyon* (SRR628921, SRR629088, SRR629207) and *Oryzasativa* (SRR1035998, SRR1035999, SRR1036000) RNAseq and BSseq were also generated from young leaf tissue. Finally, *Z. mays* (SRR850328) data were generated from seedling tissue. The differing tissues used in this study should have little effect, as methylation typically varies little between tissues (Schmitz et al. 2013; Roessler et al. 2016). These data were chosen to make our mCHH island results comparable to gbM results from the same species, using the same data and reference genomes (Seymour and Gaut 2020). Genome sizes in table 1 for each of the species were from the Kew *C*-value database (http://data.kew.org/cvalues/, last accessed September 2, 2019) except for that of *Phyllostachysheterocycla*, which came from Peng et al. (2013).

**Table 1:** A List of Species Examined in This Study, with Their Genome Size, the Number of Genes Used in Analyses and Information about CHH-Island Characteristics

Species	Genome Size (Mb)^a^	**No. of Genes** ^b^	**% mCHH Island Genes** ^c^	**Median Island mCHH Level** ^d^	**% mCHH Island Orthologs** ^e^	**Median Ortholog Island mCHH Level** ^f^
*Brachypodium distachyon*	355	34,257	55.16%	31.49%	58.20%	32.76%
*Hordeum vulgare*	5,428	35,200	28.22%	34.19%	41.34%	34.64%
*Oryza sativa*	489	41,806	71.85%	41.80%	76.61%	49.19%
*Phyllostyachys heterocyla*	2,075	30,946	17.27%	29.03%	18.51%	28.57%
*Setaria italica*	513	34,170	29.80%	38.89%	34.31%	41.00%
*Sorghum bicolor*	734	33,972	54.01%	46.05%	59.91%	49.06%
*Triticum urartu*	4,817	33,612	22.94%	34.78%	28.29%	35.71%
*Zea mays*	2,655	37,534	30.85%	53.85%	38.73%	53.80%

a Genome sizes estimated by flow cytometry, primarily from the Kew *C*-values database (see Materials and Methods).

b Number of genes used in genome-wide summaries in figures 1 and 2, including only genes with near-gene BSseq coverage (see Materials and Methods).

c The percentage of genes associated with mCHH islands within the flanking 5′ or 3′ 2.0 kb.

d The median level of CHH methylation in islands within 2 kb of genes.

e The percent of orthologs, of 2,720 total, associated with a 5′ mCHH island in each species.

f Median mCHH level for islands associated with orthologs.

For all eight species, we used methylome data provided by Seymour and Gaut (2020). Briefly, they trimmed BSseq reads for quality and adapter sequences using trimmomatic (v0.35) and used Bismark (v0.15.0) with bowtie2 (v 2.2.7) to align trimmed reads to the reference genomes of each species, with seed parameters of -N 0 -L 20. After alignment, Bismark methylation extractor (0.15.0) was used to determine numbers of methylated and unmethylated reads at each cytosine site. The accessions used in this study were the same as those used to generate the reference genomes. The reference genomes were: *S. italica* (Bennetzen et al. 2012; Sitalica_312_v2.fa), *O. sativa* (International Rice Genome Sequencing Project 2005), *Z. mays* (Schnable et al. 2009), *T. urartu* (Ling et al. 2013), *S. bicolor* (Paterson et al. 2009), *P. heterocycla* (Peng et al. 2013), *B. distachyon* (International Brachypodium Initiative 2010), and *H. vulgare* (Mascher et al. 2017). Coverage information for methylomes can be found in supplementary table S6, Supplementary Material online.

### Measuring mCHH in Windows and Defining mCHH Islands

We calculated the weighted mCHH level of defined genomic windows using a custom R script (R version 3.5.1). Following Schultz et al. (2012), Schmitz et al. (2013), the weighted methylation of a window was calculated separately for each cytosine context (CG, CHG or CHH) as the number of methylated reads in that window divided by the number of unmethylated reads at cytosines in the same context. We applied this metric to windows of various lengths for different analyses (see text). When this metric was compared with randomly chosen windows (e.g., figs. 1B and 3), we identified those windows using the sample_n() function in the R package dplyr v1.0.2 (Wickham et al. 2020).

**Fig. 1:**
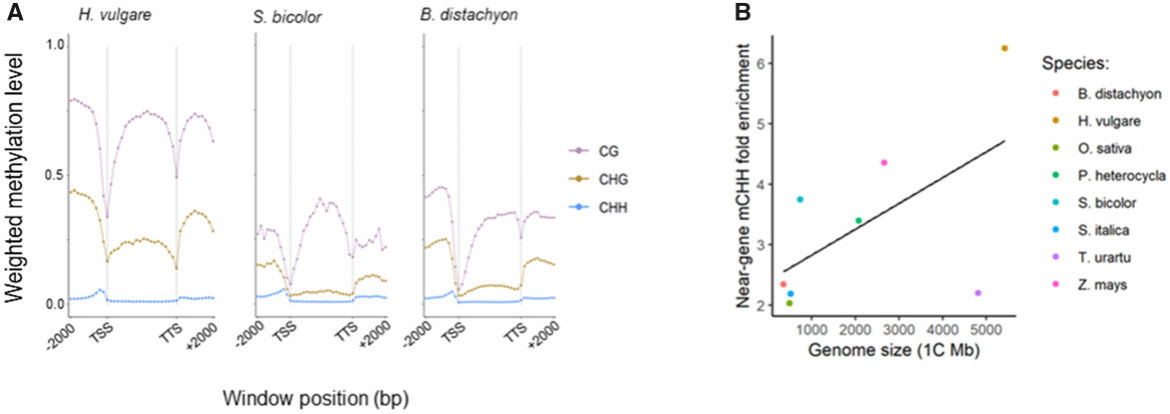
Near-gene methylation across Poaceae species. (*A*) Profiles of methylation across genes and their 2.0 kb 5′ and 3′ flanking regions. Weighted methylation levels are summarized in 10 200 bp windows upstream and downstream of genes, and in 20 equally sized windows within genes that vary in size depending on gene length. These figures summarize across full genes, with exons and introns. Here we show three species that span the range of genome size (table 1), with the remaining species shown in supplementary figure S2, Supplementary Material online. TSS and TS refer to the transcription start and termination sites. (*B*) Near-gene enrichment of mCHH increases with genome size. Near-gene mCHH enrichment represents the mean weighted mCHH levels in 1 kb regions upstream of the TSS divided by the mean weighted mCHH levels in an equal number of 1 kb regions randomly selected throughout the genome.

mCHH islands were identified using the method of Li et al (2015), altered to be applicable across species with varying genome-wide mCHH levels. Each chromosome was divided into nonoverlapping 100 bp windows and weighted methylation levels were calculated for each window. Each window was then assigned a *P* value with a one-sided binomial test for mCHH hypermethylation, similar to the method of Takuno and Gaut (2012) for genes. The 100 bp windows were annotated as mCHH islands if they were within 2 kb of gene TSS, contained more than five mCHH cytosines, and possessed an Benjamini–Yekutieli FDR-corrected *P* value < 0.01. Coverage across CHH residues was counted in the near-gene (2 kb 5′ of Transcription Start Site) region for each gene; genes were excluded if they lacked WGBS CHH site coverage >2× in more than half of this region.

For completeness, we performed the same analysis based on methylation levels calculated as the fraction of methylated cytosines (Schultz et al. 2012). In this variation, each cytosine in the CHH context was determined to be either methylated or not based on the binomial test (Lister et al. 2008) when the site had two or more reads. Genome-wide and window-wide methylation were then calculated as the percentage of cytosines that were methylated among cytosines with sufficient data. The two methods (weighted vs. per site methylation) yielded nearly identical results; for example, >95% of genes in *Z. mays* had the same designation as island or nonislands genes. All downstream analyses were qualitatively identical for the two analytical methods; we report only weighted methylation levels for simplicity.

Similarly, to ground truth our binomial test, we also explored analyses without using the binomial—that is, by employing the empirical 25% cutoff (Li et al. 2015). We found similar results between the two methods. For example, we asked what proportion of the 21,760 (=2,720 orthologs × 8 species) genes had mCHH islands based on the binomial method and the 25% cutoff. The two methods agreed for 85.8% of the genes. These results strongly suggest that our overall results are robust to some variation in the mCHH island detection approach. As a further test of robustness, we applied linear regression and variable importance analysis to mCHH islands detected with 25% cutoffs, yielding qualitatively similar results.

### Expression Analyses

We used the expression information calculated by Seymour and Gaut (2020) from RNAseq data to evaluate expression of mCHH island genes. RNAseq data came from the same tissues and accessions as BSseq data. The raw RNAseq data were filtered for quality and adapter trimming with trimmomatic (v0.35), requiring 45 bp read lengths after trimming. Alignments to reference annotations were performed using bwa (v0.7.12) allowing two mismatches (-n 2). Raw read counts were normalized (TMM) in edgeR (v3.20.9) for each species and reads per kilobase mapped (RPKM) was estimated from the fitted values. Trimmed reads were aligned to annotations available for each genome and reported in supplementary table S4 of Seymour and Gaut (2020).

Expressed genes were divided into quartiles of expression based on log_2_ RPKM using the quantile() function in R. Genes that were not present in RNA-seq data were marked as being in quartile 0. Metaprofiles showing near-gene mCHH at different expression quartiles were generated by demarcating 100 bp windows across the 2 kb regions 5′ to the TSS and separately calculating mCHH means per window for each expression quartile. To compare expression of orthologs between species, expression in RPKM was normalized to zero-mean unit variance using the scale() function in R.

### Gene Characteristics and Regression Analyses

We used phylogenetic generalized least squares (PGLS) regression to query the relationship between genome size (log 10 1C) and levels of CHH DNA methylation. PGLS regression corrects for phylogenetic relationships and requires information about branch lengths between species. For the latter, we used a phylogenetic tree inferred by Seymour and Gaut (2020) from 2,982 single-copy orthologs across the eight species of interest (supplementary fig. S1, Supplementary Material online). The single-copy orthologs were identified using orthomcl (v2.0.9) and BLASTP (v.2.2.30), with the “-evalue 1e-5 - outfmt 6” options. The phylogeny was inferred from concatenated nucleotide alignments of orthologs using ape (v5.2) and phangorn (v2.4.0) using a GTR substitution model. PGLS regression using these branch lengths was performed using nlme (v3.1.131) in R. 

We downloaded repeat annotations for all eight species (supplementary table S5, Supplementary Material online). Given annotations, we calculated the TE distance to a gene by taking the absolute value of the difference between the TSS of each gene and the 5′ or 3′ edge (whichever was closest) of the nearest TE (indiscriminate of strand). Genes without a detectable TE upstream, which were generally the first or last genes on a scaffold, were not included in this analysis. The distance was marked as zero when a TE overlapped with a gene.

We also calculated genic parameters. Gene length included both introns and exons and was calculated by subtracting the minimum from maximum annotated chromosomal position for each gene. As a comparison to gene expression, we divided genes into quartiles of length using the same method as described above for gene expression data. Weighted exonic mCG levels were calculated as before (#mCG reads/# total reads) inside exons of the longest transcript in each gene. Logistic regression models were built in R using the glm() function, using genic variables (expression, distance to a TE, length, and gbM) to predict island association as a qualitative, binary variable. We standardized each variable to a 0–1 scale by subtracting the lowest value of each set from all values in each, then dividing by the highest value in each set. We built this model separately using both gbM as a qualitative and quantitative variable, to make sure that the inclusion of a qualitative variable did not affect the outcome. We evaluated the contribution of each predictor variable to the model using the varImp() function in the caret package (Kuhn 2020).

We assessed conservation of mCHH island by focusing on the list of orthologs identified by Seymour and Gaut (2020). After filtering for near-gene coverage in our WGBS, we included 2,720 genes (supplementary table S7, Supplementary Material online). We calculated fold enrichment of mCHH island and gbM conservation by comparing observed and expected counts between pairs of species. The observed was the number of orthologs that were mCHH island associated or gbM within both species; the expected was the product of proportions of mCHH island orthologs (table 1) between the two species in each pair. We modeled the relationship between mCHH island frequency (counts from 0 to 8 across species) and each genic variable using the lm() function in R. The Feltz and Miller test (1996) was used to assess the equivalence of CVs between distances to TE edges and genes.

### BLAST Analyses

To investigate the homology of mCHH island sequences in maize, rice, and barley to TEs, we built a reference TE database. The data set consisted of: 1) *O. sativa* TE fasta files from the Rice Transposable Element Database (http://www.genome.arizona.edu/rite/, last accessed February 10, 2020) (Copetti et al. 2015), 2) *Z. mays* and *H. vulgare* TEs (Wicker et al. 2017) extracted from their reference genomes using samtools, 3) full-length TEs from Stitzer et al. (2019), accessed at https://github.com/mcstitzer/maize_TEs/blob/master/B73.structuralTEv2.fulllength.2018-09-19.gff3.gz(last accessed October 6, 2020) (Stitzer et al. 2019), and 4) repeat sequences from the Transposable Element Platform (TREP) database (Wicker et al. 2002) (https://botserv2.uzh.ch/kelldata/trep-db/index.html, last accessed April 1, 2021). This TE reference database was used as a reference with mCHH island sequences. The island sequences were run through BLASTn (v2.8.1) (Altschul et al. 1990) using discontiguous megablast (-task dc-megablast) against a custom reference fasta file containing the combined TE sequences in the database. To identify a set of random, “control” sequences, we sampled a number of non-mCHH island 100 bp windows equal to the number of mCHH islands from each genome using the sample_n() function in the R package dplyr v1.0.2 (Wickham et al. 2020). These sequences were BLASTed against the TE reference in the same manner. Sequences with no BLAST hit were assigned an *e*-value of 1.0.

## Results

### General Patterns of CHH Methylation Near Genes

We analyzed the near-gene distributions of cytosine methylation in a data set of WGBS and RNA-seq data from leaf and shoot tissue of eight grass species (Seymour and Gaut 2020) (supplementary fig. S1, Supplementary Material online; see Materials and Methods). These species represent most of the evolutionary breadth of the Poaceae and span a 15-fold range of genome sizes from 5,428 (*H.vulgare*) to 355 Mb (*B.distachyon*) (table 1). We first examined methylation in and near genes by measuring the weighted methylation level across all genes with available flanking data for 2 kb both up and down streams. Following precedent (Schultz et al 2012; see Materials and Methods), we defined the weighted methylation level of a region as the proportion of methylated versus unmethylated bases that align to a single site in the appropriate context, and then averaged across all such sites in a defined region or window. We applied this approach to plot CG, CHG, and CHH methylation in 200 bp windows and merged the results across genes (fig. 1*A* and supplementary fig. S2, Supplementary Material online). These analyses revealed well-known patterns—for example, CG methylation within genes predominated over CHG and CHH methylation, and methylation was relatively low near both transcription start sites (TSS) and downstream of transcription termination sites (TTS) (Feng et al. 2010; Zemach et al. 2010b) (fig. 1*A* and supplementary fig. S2, Supplementary Material online).

These plots also demonstrated that peaks of CHH methylation within most species are located immediately upstream to the TSS and downstream of the TTS (fig. 1*A* and supplementary fig. S2, Supplementary Material online). Four features of the mCHH peaks merit further comment. First, the peaks were identifiable despite the fact that these figures average over all genes, not just the genes with mCHH islands. Hence, the peaks likely underestimate the magnitude of methylation levels for the subset of genes that are associated with mCHH islands. Second, the CHH peaks varied in magnitude. They were most prominent in *Z. mays* (supplementary fig. S2, Supplementary Material online), suggesting either that *Z. mays* had a higher proportion of genes with mCHH islands than other species or that its mCHH islands were more highly methylated. However, mCHH peaks were also notable in *H. vulgare*, which has the largest genome in our sample, in *B. distachyon*, with the smallest genome in our sample, and in *S. bicolor*, with an intermediate size genome (fig. 1*A*). In *O.sativa*, another species with a small genome (table 1), the peak height was also pronounced, reaching >10% of methylated reads across all cytosines in the CHH context (supplementary fig. S2, Supplementary Material online). Third—as previously found in *Z. mays* (Gent et al. 2013)—mCHH peaks were far more evident in 5′ upstream regions compared with 3′ downstream regions; accordingly, most of our subsequent analyses focus on 5′ islands. Finally, the analyses of *T.urartu* and *P.heterocycla* yielded the least obvious 5′ bumps in mCHH levels (supplementary fig. S2, Supplementary Material online). Genome-wide analyses of *T. urartu* genes also yielded nonstandard patterns of genic methylation (supplementary fig. S2, Supplementary Material online). In this context, it is worth noting that these two genomes had the lowest contiguity among our sample (supplementary table S1, Supplementary Material online). In theory, low contiguity should not affect our results, because we only analyzed genes that had 2.0 kb flanking regions. However, the potential effects of fragmented data and/or poor annotations for these two species must be kept in mind.

One argument about mCHH islands is that they separate euchromatin from heterochromatin. If true, a simple prediction is that mCHH levels should be higher in species with large genomes, because they are more likely to have a high density of TEs interspersed with genic regions. To assess the relationship between mCHH levels and genome size, we measured weighted mCHH methylation in 1.0 kb regions upstream of TSS and downstream of TTS across all genes. We focused on 1.0 kb regions because this distance usually encompassed near-genic CHH peaks (fig. 1*A* and supplementary S2, Supplementary Material online). Following Gent et al. (2013), we then estimated the fold enrichment of those 1.0 kb regions by comparing them to an equal number of randomly determined 1.0 kb sites across each genome. All eight species exhibited greater than 2-fold enrichments of near-gene mCHH, ranging from 2.34× enrichment near genes in *B. distachyon* to 6.25× enrichment in *H. vulgare*. We tested the relationship between genome size and near-gene mCHH enrichment using PGLS regression (Symonds and Blomberg 2014). The relationship was not significant with all eight species (*P *=* *0.157), but *T. urartu* was a clear outlier. When we performed a post hoc analysis without *T. urartu*, the remaining seven species represented a strongly positive correlation between genome size and near-gene mCHH enrichment (*P *=* *4e-4).

To probe this result further, we examined the relationship between genome size separately with levels of CHH methylation in near-gene 1.0 kb windows (supplementary fig. S3*A*, Supplementary Material online) versus randomly chosen windows from throughout the genome (supplementary fig. S3*B*, Supplementary Material online). Near-gene mCHH levels had a slight but nonsignificant negative relationship with genome size (supplementary fig. S3*A*, Supplementary Material online) (*r*^2^ = −0.04; *P *=* *0.43). In contrast, random genomic windows had a stronger negative relationship with genome size (*r*^2^ = −0.1; *P *=* *0.22), mirroring a previous study that measured genome-wide mCHH levels in this same sample of eight species (Seymour and Gaut 2020). Putting these results together, they suggest that any relationship between genome size and mCHH enrichment (i.e., the ratio of near-gene to random windows) reflects that background levels of CHH tend to be lower in large genomes. Thus, we find no compelling relationship between genome size and near-gene mCHH levels.

### mCHH Islands Are Methylation Islands

To characterize mCHH islands more fully, we modified the method of Li et al. (2015) by splitting each genome into nonoverlapping 100 bp windows and calling windows with elevated mCHH levels as mCHH islands when they were <2.0 kb from a gene. Although Li et al. (2015) called mCHH islands using an empirical >25% mCHH cutoff, we performed a binomial test on each window to determine whether there was significantly more CHH methylation than the genome-wide level (*P < *0.01, after FDR correction) (see Materials and Methods). Note that we also applied alternative methods that either focused on the fraction of significantly methylated cytosine sites, rather than weighted methylation levels (Schultz et al. 2012), and also used an empirical cutoff rather than the binomial test (see Materials and Methods). All methods yielded qualitatively identical results with nearly identical quantitative results. For simplicity, we present the results based on weighted methylation levels, to follow the precedence of previous mCHH island analyses (Li et al. 2015; Niederhuth et al. 2016), and on the binomial test, because it is an inherently statistical approach.

The binomial method yielded information about the mCHH level of statistically identifiable islands. For example, the median mCHH level of islands was highest in *Z. mays* at 53.8%, followed by *S. bicolor* and *O. sativa*, which were between ∼40% and 50%. The remaining species all had median island levels of ∼30–40% mCHH (table 1), which is much higher than background levels of 12% or less (supplementary fig. S3*A*, Supplementary Material online). Given the identification of islands, we characterized genes as mCHH island associated (hereafter mCHH island genes) if they had at least one significantly elevated 100 bp region within 2.0 kb upstream. After examining >30,000 annotated genes per genome, we found that the proportion of island genes varied widely between species, from 17.3% in *P. heterocycla* to 71.9% in *O. sativa*, with an average of 38.8% across all eight species (table 1).

**Fig. 2:**
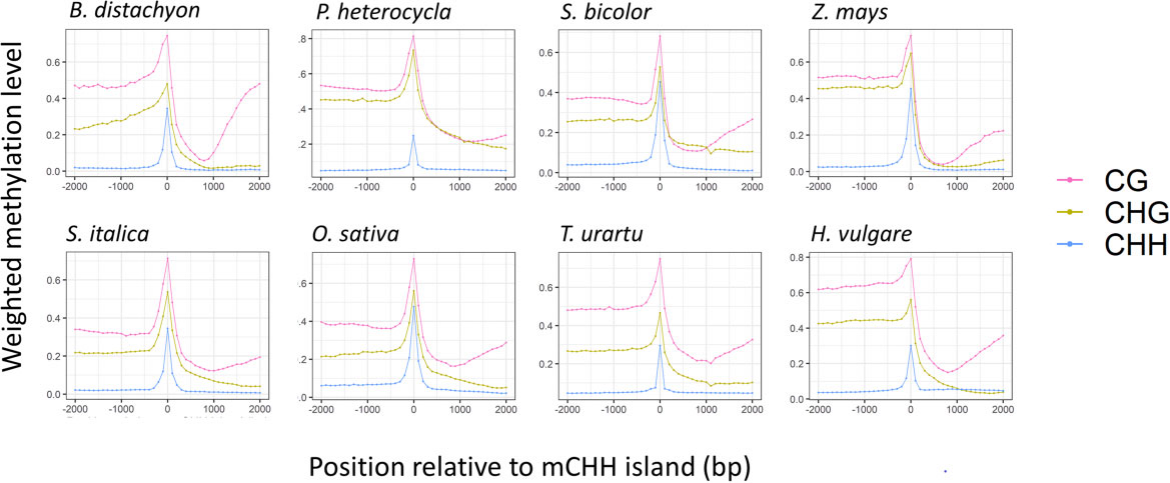
Profiles of methylation across mCHH islands in each sequence context. The *x* axis provides the distance in base pairs (bp) from a detected island, which is centered at zero. The points on the graph represent weighted methylation levels in 100 bp windows. The islands were not at a fixed distance from genes, because they were determined by significance tests, but they were within the 2 kb 5′ flanking region of genes.

To assess methylation levels around mCHH islands, we focused on the center of a single 5′ 100 bp mCHH island window and plotted the average upstream and downstream of that center. As expected, we found that methylation distributions were elevated in the CHH context, but the results showed that islands were also elevated in the CG and CHG contexts in all eight species (fig. 2). Thus, as noticed previously (Niederhuth et al. 2016), mCHH islands are really “methylation islands,” because they contain elevated methylation levels in all three cytosine contexts. This result further reinforces previous conclusions that mCHH islands represent RdDM deposition (Gent et al. 2013; Li et al. 2015), because RdDM is agnostic with respect to cytosine context (Matzke and Mosher 2014).

### Genic Attributes of mCHH Island-Associated Genes

mCHH islands are hypothesized to function as a boundary between TE-enriched heterochromatin and gene-rich euchromatin (Gent et al. 2013). This hypothesis predicts that island genes should be adjacent to TE-rich regions more often than nonisland genes. One way to examine this prediction is to investigate genome size, but this approach does not recognize that different genomes may have different organization of TEs and genes that may not be tightly correlated with genome size. Hence, to test this prediction in more detail, we explored the relationship between CHH island genes and TEs. For each species, we first downloaded publicly available annotations of each genome (supplementary table S2, Supplementary Material online). Then, for each gene in each species, we identified the annotated repetitive element closest to the TSS and measured the distance from the gene TSS to the nearest end of the repeat. As expected given previous research (Li et al. 2015), we found that mCHH island genes are much closer to repeats, on average, than nonisland genes, and this was true for each of the eight species (logistic regression; *P *<* *0.01). Although the signal was consistent across each species, note that the quality of repeat annotations likely vary across genomes, as does genome quality.

Another attribute of maize mCHH islands was their association with gene expression (Gent et al. 2013; Li et al. 2015), but the relationship between islands and gene expression did not hold across angiosperms in a more extensive data set (Niederhuth et al. 2016). We reinvestigated this relationship on a smaller scale by first repeating the analyses of previous studies (Gent et al. 2013; Li et al. 2015; Niederhuth et al. 2016). These studies separated genes into quartiles of expression and plotted mCHH levels upstream of genes. Our results were similar to previous work, showing that more highly expressed genes were slightly enriched for mCHH in all species (fig. 4*A* and supplementary fig. S4, Supplementary Material online). We also contrasted expression differences between mCHH island versus nonisland genes (fig. 4*B*). In all eight species, mCHH island genes had slightly higher average expression levels than nonisland genes, but the difference was significant for only three species (*Z. mays*, *H. vulgare*, and *P. heterocycla*). These results mimic Niederhuth et al. (2016) by suggesting that a relationship between mCHH islands and gene expression is either not universal or that it is so subtle as to be difficult to support statistically in some species.

We investigated additional genic features that may be associated with mCHH islands. For example, Li et al. (2015) reported a small (but nonsignificant) enrichment of gene-body methylation (gbM) genes among mCHH island genes. We assessed the relationship between mCHH islands and gbM in two ways, using gbM either as a binary trait or as a quantitative variable (weighted mCG levels within exons) (see Materials and Methods). In both cases, we found a negative relationship between mCHH islands and genic methylation, and this negative relationship held for all species (logistic regression with %GC, *P *<* *3.9e-6 for each species). We also tested whether island-associated genes were longer than other genes, because gbM genes are typically longer than unmethylated genes (Takuno and Gaut 2012). mCHH island genes were significantly longer than nonisland genes in all species except *H. vulgare* and *P. heterocycla* (*P *<* *0.05, logistic regression) (fig. 4*D*), and this relationship held for both total gene length and length of longest transcript (supplementary fig. S6, Supplementary Material online). As a comparison to gene expression, we plotted genes by length quartiles (fig. 4*C* and supplementary fig. S5, Supplementary Material online), illustrating that the relationship with gene length is more obvious.

Finally, we incorporated all four predictors (TE distance, gene expression, gbM, and total gene length) into a logistic regression model for each species. Gene expression and gene length were positive predictors of island presence. TE distance and gbM were negative predictors and significant in all eight species (supplementary table S2, Supplementary Material online). A limitation of logistic regression is that the estimates for predictors are on different scales, so it is difficult to compare their effects directly to one another from the estimates. To circumvent this problem, we applied variable importance analysis (Kuhn 2020), which scales predictors for direct comparison within a model (fig. 5; see Materials and Methods). Three notable patterns emerged. First, TE proximity was generally—but not always—the most powerful predictor of the presence of an mCHH island. TE proximity was the most important variable in five of eight species, but gbM was the strongest predictor in the remaining three species. Second, among species, TE proximity was least important in *T. urartu*, which could again reflect features of genome or annotation quality. Third, gene length was also consistently significant, but its importance was always eclipsed by gbM and TE proximity. Finally, gene expression was comparatively unimportant, even in the three species for which it was a significant predictor (supplementary table S2, Supplementary Material online).

### Assessing Evolutionary Conservation of mCHH Islands

The availability of a set of orthologs from these species facilitates the address of another question: are mCHH islands conserved over evolutionary time? To address this question, we investigated mCHH island conservation among 2,720 orthologs (Seymour and Gaut 2020). Islands were recorded as a binary trait for each ortholog; that is, each gene was or was not associated with an island in each species (table 1). We then contrasted pairs of species and calculated the enrichment of island conservation. Enrichment was measured as the ratio of the number of orthologs with conserved islands between species to the number expected at random (see Materials and Methods). mCHH islands did not exhibit a signal consistent with a signal of evolutionary conservation (fig.5*A*). Enrichment between species never exceeded 1.1× (table 1), and the number of orthologs with conserved island association was not significantly greater than expected by random chance in any pairwise comparison (permutation test, *P *>* *0.05). As a contrast, we also investigated gbM conservation, because it is an epigenetic state that is known to be conserved between orthologs from different species (Takuno and Gaut 2013; Seymour et al. 2014; Niederhuth et al. 2016; Takuno et al. 2016; Seymour and Gaut 2020). In comparison to mCHH island enrichment levels of <1.1×, gbM conservation ranged from a minimum of 2-fold enrichment to as much as 3.5× enrichment (fig. 5*A*).

We further examined some of the features that may contribute to rare cases of mCHH island conservation. We began by plotting, for each of 2,720 orthologs, the number of islands across eight species. The distribution of mCHH island conservation among orthologs (supplementary fig. S7, Supplementary Material online) had a median of four species and a mean of 3.57 species, which was statistically indistinguishable both from the expected mean of 3.55 species under a purely random model (simulation, *P *=* *0.272) and from normality (Shapiro-Wilkes test, *P > *0.05). To investigate further, we applied linear models to test for correlations between gene-associated variables and maintenance of mCHH island status over evolutionary time. For example, the average exonic %CG across orthologs in all eight species was significantly negatively correlated with the number of species that had a gene island (*r*^2^ = −0.0003, *P *=* *0.003) (fig. 5*B*). Using the same approach, we found that the average expression of an ortholog was not correlated with the number of species that have an mCHH island (*r*^2^ = 9.2–2e-4, *P *=* *0.0567, fig. 5*C*) but that average gene length was positively correlated (avg. gene length, *r*^2^ = 0.0029, *P *=* *0.011, fig. 5*D*). The largest correlation was between conservation and TE distance (*r*^2^ = −0.051, *P *=* *9.6e-21, fig. 5*E*), providing further evidence of the link between mCHH islands and TEs. Although the *r*^2^ values of these significant correlations were very low, they largely recapitulated our within-species analyses.

### mCHH Islands and TE Superfamilies in Maize, Rice, and Barley

Finally, we brought together data on mCHH islands, TEs, and orthologs to further investigate the link among mCHH islands, genes, and specific types of TEs. For these analyses, we narrowed our focus to three well-studied species—*Z. mays* (maize), *O. sativa* (rice), and *H. vulgare* (barley)—that had both reasonably contiguous genomes (supplementary table S1, Supplementary Material online) and careful TE annotations that distinguished among element superfamilies (Wicker et al. 2007; table 2).

**Table 2:** Counts of TEs within 2 kb for a Common Set of TE Superfamilies across Species and Their Enrichment Status for mCHH Islands

	Barley			Rice			Maize		
**TE Family** ^a^	**#TEs <2 kb** ^b^	**% with Island** ^c^	**Enriched** ^d^	#TEs <2 kb	% with Island	Enriched	#TEs <2 kb	% with Island	Enriched
DHH	69	0.174	NS	132	0.417	Under	5,235	0.283	Under
DTA	21	0.095	NS	517	0.768	NS	653	0.542	Enriched
DTC	3,480	0.280	NS	1,243	0.474	Under	184	0.429	Enriched
DTH	478	0.460	Enriched	40	0.700	NS	2,677	0.536	Enriched
DTM	654	0.378	Enriched	1,106	0.806	Enriched	122	0.623	Enriched
DTT	474	0.430	Enriched	2,143	0.898	Enriched	2,307	0.389	Enriched
DTX	332	0.497	Enriched	6,476	0.882	Enriched	299	0.408	Enriched
RIX	880	0.227	Under	551	0.611	Under	87	0.253	NS
RLC	4,896	0.239	Under	1,433	0.651	Under	3,148	0.280	Under
RLG	4,413	0.213	Under	2,230	0.580	Under	3,891	0.292	Under
RLX	11,356	0.315	Enriched	1,0753	0.704	Under	2,632	0.224	Under
RSX	76	0.316	NS	796	0.932	Enriched	43	0.302	NS
**Total**	**27,129**	**0.285**		**2,7420**	**0.747**		**21,278**	**0.333**	

a TE classification code as described by Wicker et al. (2007). DHH, *Helitron*; DTA, *hAT*; DTC, *CACTA*; DTH, *PIF-Harbinger*; DTM, *Mutator*; DTT, *Tc1-Mariner*; DTX, unknown DNA elements; RIX, unclassified *LINE*; RLC, *Copia*; RLG, *Gypsy*; RLX, unclassified *LTR*; RSX, unclassified *SINE*.

b The number of TEs within each class that are within 2 kb upstream of an annotated gene, based on counting only the closest TE to a gene.

c The proportion of genes that have both an mCHH island and a TE within 2 kb upstream.

d Based on a binomial test (FDR corrected, *P* < 0.05), classes of TEs were determined to be significantly enriched (Enriched) for CHH islands or under-enriched (Under), relative to the total proportion estimated across all TE superfamilies. NS, nonsignificant.

#### mCHH Islands and Homology to TEs

If the primary function of mCHH islands is to silence near-gene TEs (Li et al. 2015), their lack of evolutionary conservation is unsurprising because TE content often varies between species. Under this model, one expects mCHH islands to be associated with sequences that have homology to TEs and perhaps to specific TE families (Zemach et al. 2010a, Li et al. 2015). Given data from maize, rice, and barley, we first counted how often TEs were 2 kb upstream of the TSS of an annotated gene and then assessed whether those genes had a 5′ mCHH island. The results varied markedly among species; ∼30% of genes had both a TE and an mCHH island in barley and maize, but 74% of genes fell into this category in rice (table 2). The interesting point about these values is that many mCHH islands—about 70% in maize and barley—are not obviously associated with nearby TEs.

**Fig. 3:**
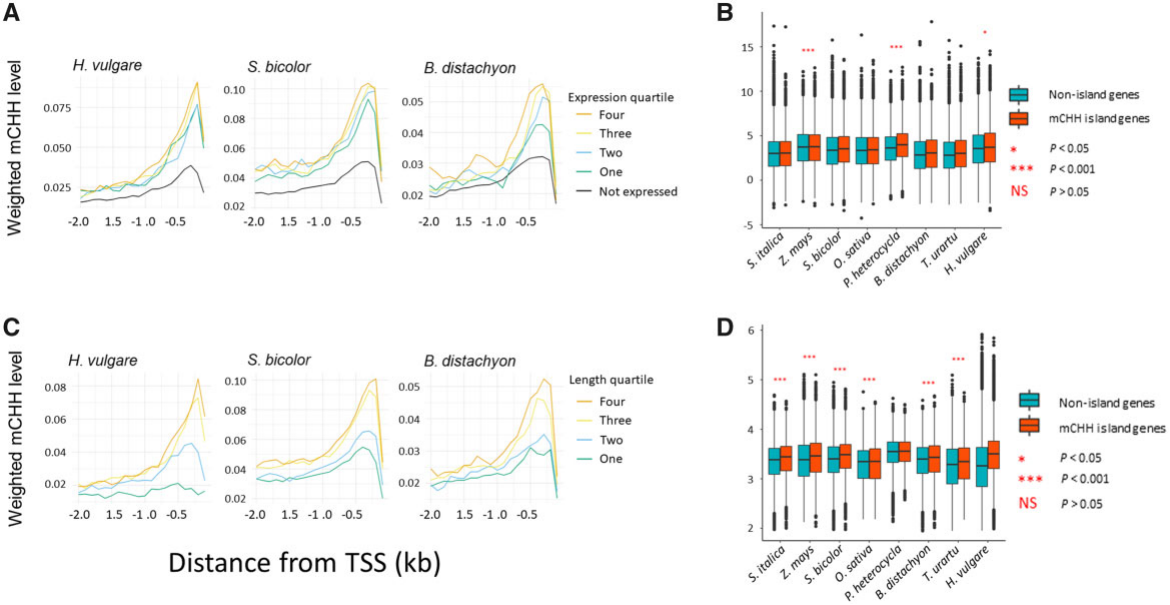
mCHH island relative to gene expression and length. (*A*) Profiles of near-gene methylation in genes separated into four quartiles of expression and into nonexpressed genes. The graphs illustrate for some species that genes in the higher quartiles tend to have higher 5′ flanking CHH methylation. (*B*) Expression levels between mCHH island genes and nonisland genes. Significance levels between the two categories are shown for each species, with NS = not significant. (*C*) Profiles of near-gene methylation in genes separated into four quartiles for gene length. (*D*) The length of island and nonisland genes. Significance levels between the two categories are shown for each species, with NS = not significant. These length measures were based on distances from the TSS to the TTS, but the results hold using the length of exons in the longest transcript (supplementary fig. S6, Supplementary Material online). For panels (*A*) and (*C*), the species were chosen because they represent a range of genome size, as in figure 1. The remaining species are shown in supplementary figures S4 and S5, Supplementary Material online. For (*B*) and (*D*), the box plots present the median, with the edges representing the upper and lower quartiles.

One likely possibility for the low overlap with annotated TEs is incomplete annotations, particularly if mCHH island sequences are within fragmented remnants of TEs. To investigate further, we aligned mCHH island DNA sequences to a database of annotated TE sequences from Poaceae genomes using BLAST, and tallied the *e*-values of mCHH island sequences (see Materials and Methods). As expected, a large proportion of island sequences had high-threshold hits to TEs—for example, 65.8%, 72.0%, and 82.0% of island sequences had homology to TEs at an *e*-value < 1e-5 in *Z. mays*, *O. sativa*, and *H. vulgare*, respectively. Nonetheless, this implies that from 18.0% to 34.2% of sequences had little homology to TEs. As a genome-wide comparison for context, we sampled the same number of random 100 bp regions from throughout each genome and mapped them to the TE database. In the case of *Z. mays* and *H. vulgare* (fig. 6*A*), a smaller proportion of mCHH island sequences had significant (<1e-5) sequence homology to TEs than the random regions (65.8% vs. 78.9% in *Z. mays*; 82.0% vs 86.2% in *H. vulgare*). Moreover, in both species there was a substantial dearth of mCHH island sequences with exact (*e*-value < 1e-40) hits to annotated TEs. The situation differed somewhat in *O. sativa*, because it had a greater proportion of mCHH islands (72.0%) with <1e-5 *e*-values compared with control regions (55.1%), but it again had a lower proportion of islands with stringent hits (*e*-value < 1e-40) (fig. 3*A*). Overall, these results indicate: 1) that a substantial proportion of mCHH islands were not obviously derived from TEs, and 2) when they did exhibit homology to TEs, they were often diverged such that they did not have especially stringent matches.

#### Associations with Specific TE Superfamilies

Both Zemach et al. (2010a) and Li et al (2015) found especially strong signals of association between mCHH islands and TIR DNA transposons. We therefore investigated particular classifications of TEs, asking whether their presence within 2 kb of a gene led to mCHH enrichment. We performed this analysis for 12 TE classifications (table 2) that were present in all three species. The enriched TE types varied among species, but there was a clear general trend: DNA transposons tended to be enriched for mCHH islands and retrotransposons were not (table 2). For each 5′ mCHH island within an annotated TE, we also measured the distance to the closest 5′ or 3′ end of the TE and the distance to the TSS of the gene (fig. 6*B*). By definition, the mean distance of within-TE mCHH islands to the edge of the TE was smaller than the distance to the TSS. Surprisingly, however, the coefficient of variation (CV) of distance to the TSS was always smaller than the CV of the distance from the mCHH island to the TE end; this was true for every TE classification and species (supplementary table S3, Supplementary Material online and fig. 6*B*;*P* ≅ 0, Feltz and Miller asymptotic test for equality). Assuming the TE annotations were accurate, these results suggest that the location of islands are influenced by their position relative to genes more than their location within TEs.

#### mCHH Islands and TEs between Orthologs

**Fig. 4:**
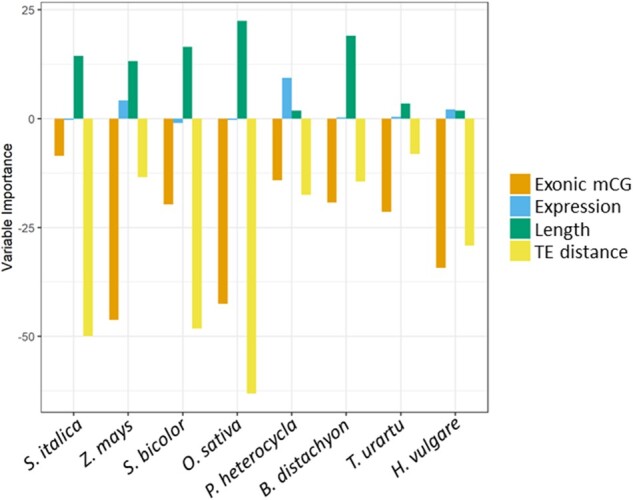
Variable importance analysis of the logistic regression model presenting the contribution of each variable to the model on an equivalent scale. Values <0 on the *y* axis denote a negative association between the predictor and the presence of a CHH island; values >0 are positive predictors.

If TE movement contributes to low conservation of mCHH islands between orthologs, the presence/absence of a TE should frequently coincide with the presence/absence of an mCHH island between species. We leveraged the set of 2,720 orthologous genes for *Z. mays*, *O. sativa*, and *H. vulgare* to test this idea. For each ortholog, we examined the presence or absence of mCHH islands between two species and then evaluated whether the orthologs had a TE within 2 kb. Focusing on orthologs that had lineage-specific mCHH islands (i.e., an island in only one of the two species), we determined whether the mCHH island was “dissonant” or “coincident” with the TE, as defined in figure 6*C*. As expected from our within-genome analyses (fig. 4), the presence of an mCHH island often corresponded with the presence of a TE, because coincident events were more frequent than dissonant events for each of the three species contrasts (χ^2^; *P *<* *0.006). The effect also varied by TE types, because coincident lineage-specific mCHH islands were: 1) significantly overrepresented for DTH (*Harbinger*) transposons and 2) significantly underrepresented for RLC (*Copia*), RLG (*Gypsy*), and DHH (*Helitrons*) (supplementary table S4, Supplementary Material online). Overall, the cross-species comparisons support inferences based on within-species data (table 2) by suggesting that TEs—and specific TE superfamilies—are associated with mCHH islands.

## Discussion

We have identified mCHH islands across a sample of eight grass species and documented their patterns relative to genome structure and function. Our study agrees with previous work by showing that mCHH islands have elevated methylation in all three sequence contexts (Niederhuth 2016), that they vary in prevalence across species (Niederhuth 2016), and that they tend to be associated with TEs (Zemach et al. 2010b; Li et al. 2015). Our work complements and confirms previous work, but it also provides novel insights into the evolutionary dynamics of mCHH islands as well as associations between mCHH islands and features of nearby genes.

### TEs Are Associated with, but Not Sufficient to Explain, mCHH Islands

Because mCHH islands in maize may act as a boundary between euchromatin and heterochromatin (Gent et al. 2013; Li et al. 2015), we predicted that the prevalence and level of mCHH islands varies with genome size, because larger genomes have more TEs (Tenaillon et al. 2010) and presumably more heterochromatin. We tested the relationship between mCHH islands and genome size in a few ways. We first examined levels of CHH methylation near genes against randomly chosen background windows of similar size. Although we could recapitulate a modest negative correlation between background mCHH levels and genome size (Seymour and Gaut 2020), near-gene mCHH levels were not correlated with genome size (supplementary fig. S3, Supplementary Material online). The ratio of these two measures—that is, the enrichment of mCHH levels near genes relative to the background—was positively correlated with genome size when *T. urartu* was not considered. To the extent that these enrichment analyses are accurate, it appears to be driven by the fact that larger genomes have lower genome-wide mCHH levels. We suspect this negative correlation reflects that larger genomes have a higher proportion of deeply silenced heterochromatin, which is typically not targeted by RdDM for de novo CHH methylation (Zemach et al. 2013).

Separately, we leveraged our mCHH island annotations to measure the median mCHH level of mCHH islands in each species and to identify the proportion of genes across the genome that have an mCHH island within 2.0 kb upstream of their TSS. Neither of these values were obviously positively associated with genome size (table 1); if anything, small genomes tended to have higher (although nonsignificant; *r*^2^ = −0.63; *P *=* *0.09) proportions of genes associated with islands. The higher proportions in smaller (and more densely CHH methylated, supplementary fig. S3*B*, Supplementary Material online) genomes are particularly notable given the biases in our statistical approach (see Materials and Methods), which favors identification of islands in larger genomes with lower CHH background methylation levels. Ultimately, the evidence for a relationship between genome size and mCHH islands remains ambiguous: larger genomes have lower background mCHH levels and thus experience somewhat higher near-gene mCHH enrichment, but smaller genomes tend to have a higher proportion of genes with mCHH islands.

Failing to find any compelling relationships with genome size, we turned to genome architecture and particularly to the potential association between mCHH islands and TEs. Consistent with previous work, we find that the presence of a nearby 5′ repeat is a significant predictor of the presence of an mCHH island (Niederhuth et al. 2016). We also focused more carefully on three species—maize, rice, and barley—that have well-established TE annotations, allowing us to assess whether specific TE classes and superfamilies are particularly associated with mCHH islands. Similar to previous studies of rice and maize (Zemach et al. 2010b, Li et al. 2015), mCHH islands are most consistently associated with TIR DNA transposons across species (table 2). The details do vary somewhat because some TIR superfamilies like DTA (*hAT* elements) are associated with mCHH islands in maize but not significantly so in rice and barley. Nonetheless, TIR elements contrast markedly with retrotransposons, which are usually not enriched for CHH island associations (table 2). It is worth noting that our method to test for enrichment only considers elements within 2 kb of a gene. Thus, these results do not simply reflect that most retrotransposons are located far from genes; when they are close to genes, they are associated with an mCHH island less often than DNA elements.

**Fig. 5:**
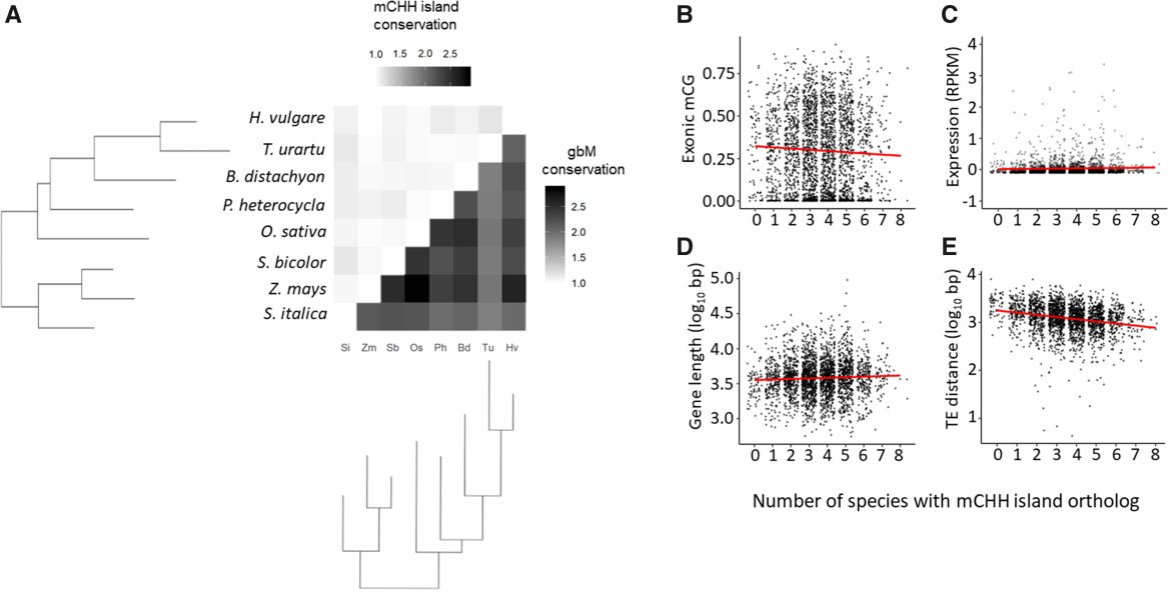
Conservation of mCHH islands across orthologs in grass species. (*A*) A heatmap of the enrichment of features over the random expectation of 1.0. Top half: enrichment of mCHH island conservation between pairs of species based on one-to-one orthologs. Bottom half: enrichment of gbM between pairs of species based on one-to-one orthologs. (*B*–*E*) Graphs of the relationship between mCHH island conservation and each genic predictor variable: exonic mCG level (*B*), expression (*C*), length (*D*), and TE distance (*E*). For each graph, the *x* axis denotes the number of orthologs, of eight total, with a 5′ mCHH island, and the *y* axis denotes the average value of the stated statistics in the ortholog across species.

**Fig. 6:**
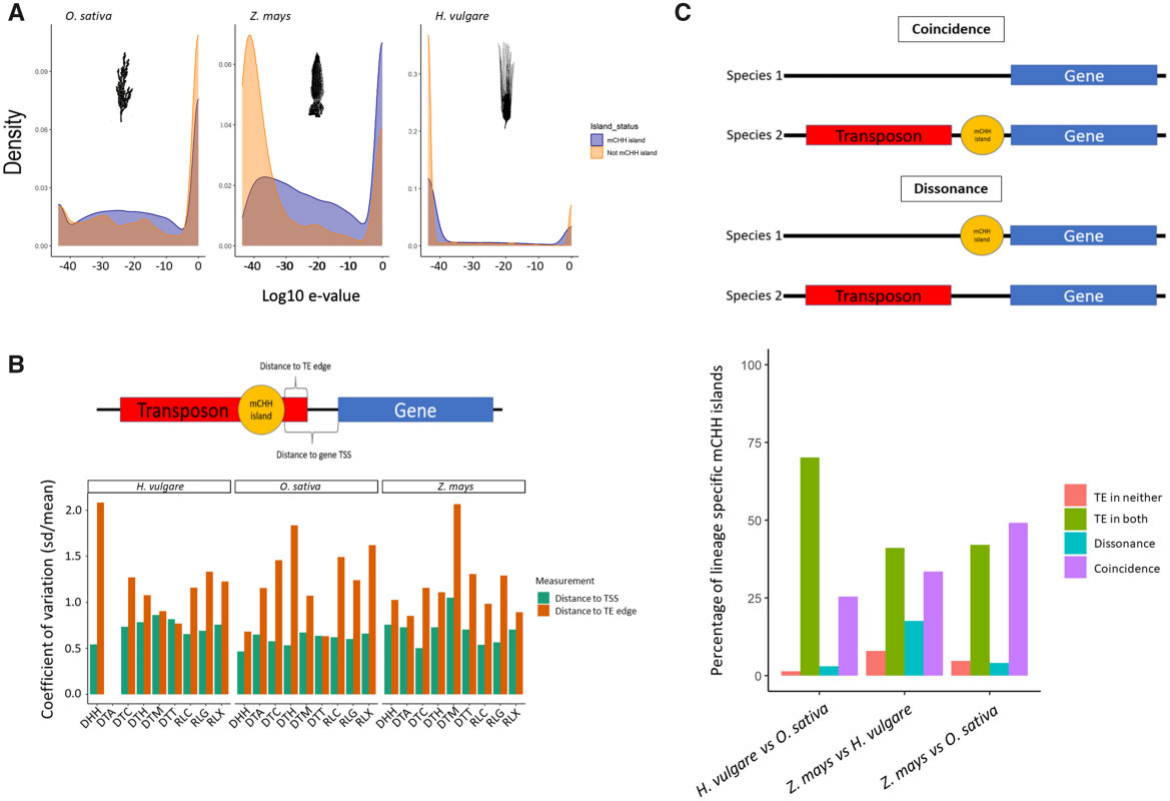
**—**mCHH islands in relation to TE presence. (*A*) The distribution of *e*-values after blasting sequences to an annotated TE database for *Z. mays* (left) and *O. sativa* (middle) and *H. vulgare* (right). Each graph plots the results for 100 bp mCHH island DNA sequences and an equal number of randomly chosen 100 bp nonisland sequences for comparison. (*B*) The coefficients of variation for mCHH island distances from gene TSS (orange) and TE edges (green) for each of the different types of TEs analyzed (Wicker et al. 2007). The schematic above the graphs defines the distances measured. (*C*) A schematic that defines the use of the terms coincident and dissonant. Each term describes a comparison of orthologs between pairs of species, with a lineage-specific 5′ mCHH island in only one species. Coincidence is when there is a lineage-specific TE and island in the same species; Dissonance is when the TE and island are in different species. The bar graph shows the frequency with which orthologs possess a lineage-specific mCHH island and the presence of TEs in neither lineage, both lineages or a single lineage (coincidence and dissonance) in the three pairwise comparisons between maize, rice, and barley.

Previous work has hinted that mCHH islands are evolutionarily labile, because only ∼64% of B73 genes had conserved mCHH enrichment (>10% mCHH) across five maize accessions (Li et al. 2015). By examining a set of 2,720 1:1 orthologs identified across all eight species (Seymour and Gaut 2020), we have shown that 5′ conservation of mCHH islands was never greater than expected by random (fig. 5*A*). However, the presence of lineage-specific TEs coincides significantly with the presence of a lineage-specific mCHH island (fig. 6*C*). TEs turnover rapidly in noncoding regions; this turnover provides at least a partial explanation for the lack of conservation of mCHH islands.

### Genic Properties Associated with mCHH Islands

Although TEs (and particularly DNA transposons) are clearly associated with the presence of mCHH islands, TEs are not sufficient to explain the presence of mCHH islands. This was illustrated aptly by Gent et al. (2013), who found that the proximal half of near-genic TEs was more highly CHH methylated than the distal half. Gent et al. (2013) ultimately concluded that mCHH islands are the product of “an interaction between genes and neighboring sequences” that can be independent of TEs. A subsequent study of maize showed that islands are enriched at the edge of transposons, particularly (TIR) elements, due to RdDM activity (Li et al. 2015). However, they also found that only ∼40% of maize mCHH islands are associated with TIR elements, again supporting the view that the TEs may are not fully sufficient to explain mCHH islands. Consistent with previous work, our analyses show that an appreciable proportion of mCHH island sequences do not have strong BLAST hits (*e*-value < 1e-5) to a TE database and that most do not have strong homology to existing TEs. Thus, many mCHH islands may not be derived from active silencing of annotated TEs.

If mCHH island sequences are not specific to a TE, what explains their presence? One possibility is that TEs trigger epigenetic modifications that then spread to adjacent chromosomal regions. If spreading occurs over sufficient distances, it could in theory explain two observations—that is, that mCHH islands often exist when a TE is not within 2 kb of a gene and that a large proportion of mCHH islands have little homology to TE sequences. Yet, mCHH islands are also clearly a function of genic properties. For example, the maize literature has established that mCHH island genes tend to be highly expressed (Gent et al. 2013, 2014; Li et al. 2015), although it has not been clear if this relationship holds across species (Niederhuth et al. 2016). We have measured gene expression in all eight species and contrasted expression levels between genes that had and did not have nearby 5′ mCHH islands. mCHH island genes are generally more highly expressed than genes without islands, but this relationship is not significant in five of eight species. Intriguingly, the three species that have a significant association have the largest genomes, an observation for which we have no ready explanation (fig. 4 and supplementary fig. S4, Supplementary Material online). There is also an important caveat: we have only examined expression in one tissue, but the tissue(s) under study may be critical, as may be expression breadth (Li et al. 2015). Future studies need to interrogate across more tissue types.

Surprisingly, in all species, gbM is a stronger predictor of mCHH islands than gene expression; in fact, gbM is even a stronger predictor than TE proximity in three of eight species (fig. 4). Our observed negative gbM relationship differs from the positive association documented previously in maize (Li et al. 2015), which examined a subset of syntenic genes. It is difficult to know whether differences between studies reflect the particular subset of genes or specific features of their data. However, we retrieve the same negative relationship when we focus only on the ortholog gene set and on alternative measures of gbM (e.g., presence/absence instead of quantitative measures). Altogether, our results show that 5′ mCHH islands are associated with genic properties that include (from stronger to weaker associations): gbM, gene length, and gene expression. Intriguingly, mCHH islands are also located at a more consistent distances from the TSS than from the edge of the TE in which they reside (fig. 6*B*), suggesting that spacing relative to the gene is more important than the physical confines of a TE.

### Additional Questions about mCHH Islands

This study has confirmed several features of mCHH islands and discovered more, but it leaves at least two important questions unanswered: how are mCHH islands formed and what is their function? We cannot answer either question, but we can provide a few additional insights. Previous work in maize has shown that the proximal mechanism of formation is RdDM (Gent et al. 2013; Li et al. 2015), which is consistent with the fact that mCHH islands have high methylation across all three methylation contexts (fig. 2). Our genome-wide results uphold the view that this is not solely a TE-driven phenomenon, suggesting again that mCHH islands represent an interaction between active genes and their neighboring sequences (Gent et al. 2013). A crucial feature of this interaction may be RNA polymerase II (Pol II) (Gent et al. 2013), because it is necessary for both genic transcription and for noncanonical (*RDR6*) RdDM (Zheng et al. 2009; Cuerda-Gil and Slotkin 2016).

The specific characteristics of genes or their neighboring sequences that trigger island formation remain unclear. Recent work has shown that maize mCHH island targets are enriched for a specific CG-rich sequence motif (Long et al. 2021; Seymour et al. 2014), but this motif neither fully explains the existence of islands nor our observations about gbM and gene length. Another possibility is that mCHH islands represent a consequence of erroneous gene transcription (Gent et al. 2013). In this model, genes occasionally experience internal and bidirectional initiation of transcription, leading to transcripts which extend beyond the 5′ end of the gene or beyond the polyadenylation site. This transcription of neighboring sequences could engage RdDM and precipitate mCHH islands, especially when those transcripts encompass nearby TEs. Once established, CHH islands may help to moderate the effects of neighboring TEs on gene expression by binding the SUVH1 and SUVH3 mediated complex (Harris et al. 2019; Raju et al. 2019).

This proposed mechanism of island formation complements one of our primary observations, which is that mCHH islands and gbM are negatively associated, because one of the presumed functions of gbM is to suppress internal transcription (Zilberman et al. 2007). Although evidence for this gbM effect is admittedly mixed (Neri et al. 2017; Teissandier and Bourchis 2017; Zilberman 2017; Le et al. 2020), it could drive the observed negative association between gbM and mCHH islands. Under this model, gbM suppresses aberrant transcription but mCHH islands result from aberrant transcription, leading to a negative association. This model is also consistent with our finding that mCHH island genes are generally longer than other genes, because longer genes have a higher probability of containing a cryptic internal promoter. The model also helps to explain the relationship between gene expression and TE proximity, because nonexpressed genes have no Pol II activity and hence could not develop islands.

Interestingly, a small proportion of genes (ranging from 5.5% in *P. heterocycla* to 26.0% in *O. sativa*) have *both* gbM and mCHH islands. This is not predicted by our model unless this subset of genes is particularly prone to aberrant transcription. We predict that such genes should be highly expressed and may represent rare cases in which the two epigenetic features are reinforcing and perhaps even synergistic. Consistent with the prediction, genes with both epigenetic features are more highly expressed than genes than with just one of the two features, and this observation holds across all eight species (supplementary fig. S8, Supplementary Material online). Although intriguing, it is at best preliminary evidence for the model that posits that both gbM and mCHH islands are related to aberrant transcription. Further analyses of aberrant transcription may prove insightful, recognizing that the effect may be subtle, just as the effects of gbM on gene expression are subtle but have become evident with the analysis of larger and more expansive data sets (Muyle et al. 2021). Another important avenue for future research will be analyses of expression breadth and responsiveness as they relate to mCHH islands.

## Supplementary Material

Supplementary data are available at *Genome Biology and Evolution* online.

## Supplementary Material

evab144_Supplementary_Data

## Data Availability

All of the data used in this article are publicly available. They are downloadable from the NCBI Short Read Archive under accession PRJNA340292, SRR628921, SRR629088, SRR629207, SRR1035998, SRR1035999, SRR1036000, and SRR850328. We have uploaded .bed files of mCHH island positions at https://figshare.com/articles/dataset/mCHH_island_bed_files/14454354.
