# Supplementary material for: CHH Methylation Islands: A Nonconserved Feature of Grass Genomes That Is Positively Associated with Transposable Elements but Negatively Associated with Gene-Body Methylation
Source: Genome Biol Evol. 2021 Jun 19;13(8):evab144. doi: 10.1093/gbe/evab144 (PMC8374106; doi:10.1093/gbe/evab144)
Supplement: evab144_Supplementary_Data [file evab144_supplementary_data.pdf]

Supplemental Figures and Tables

CHH Methylation Islands: A non-conserved feature of grass genomes that is positively associated with transposable elements but negatively associated with gene-body methylation

Galen T. Martin[1], Danelle K. Seymour[2], Brandon S. Gaut[1,*]

S1

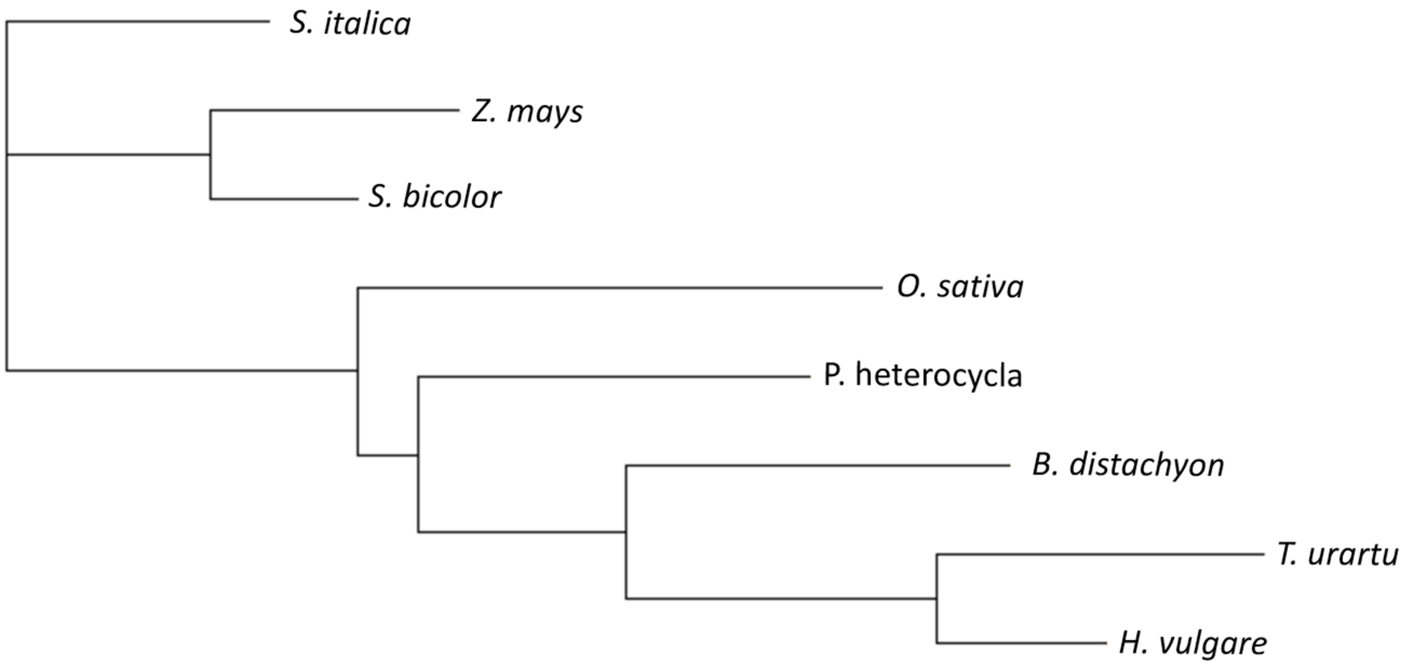

Figure S1. Maximum likelihood phylogeny of the eight Poaceae species used in this study, inferred from single-copy orthologous genes.

S2

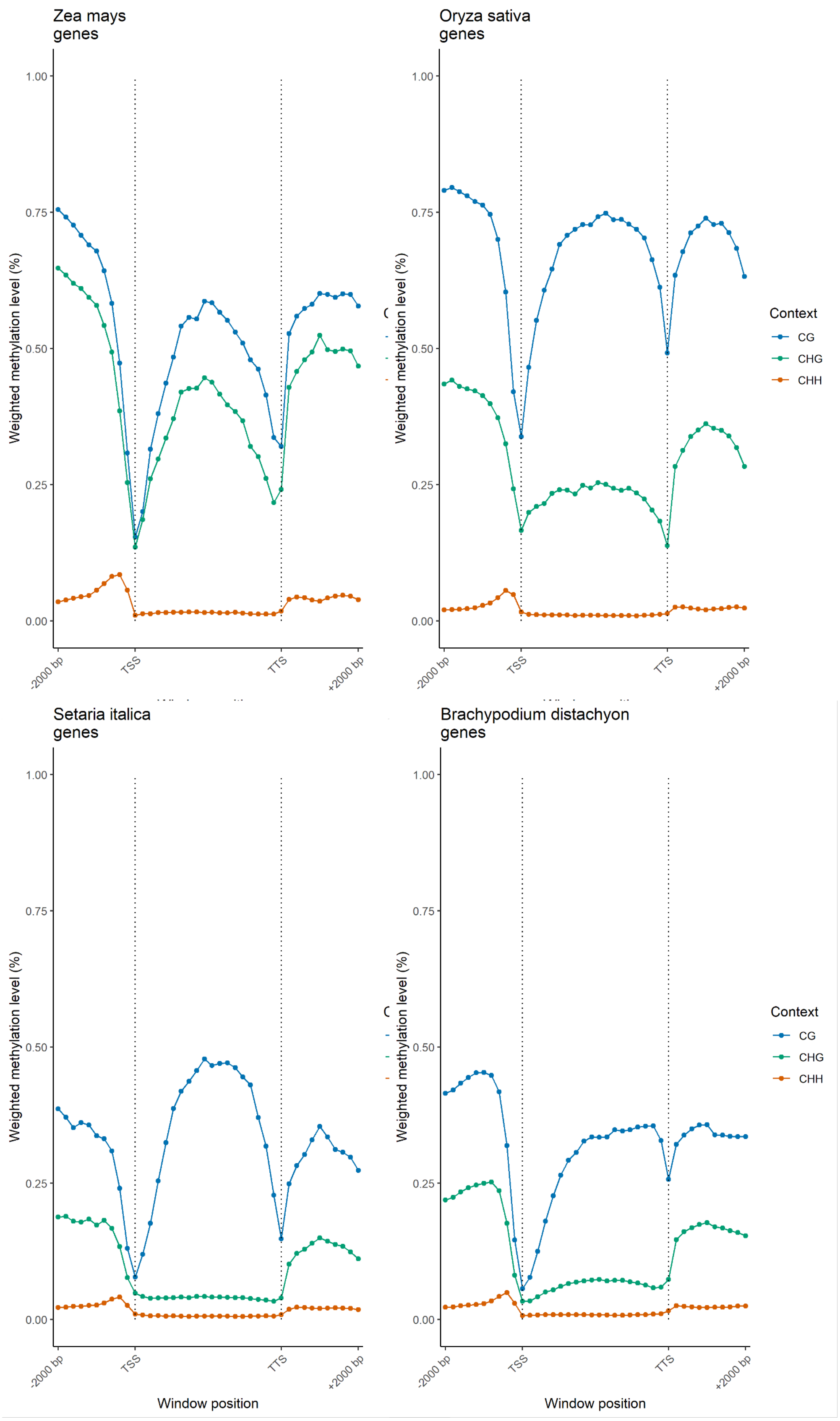
Zea mays
genes
Oryza sativa
genes
Setaria italica
genes
Brachypodium distachyon
genes
Weighted methylation level (%)
1.00
0.75
0.50
0.25
0.00
-2000 bp
TSS
TTS
+2000 bp
Window position
Context
CG
CHG
CHH

S2. continued

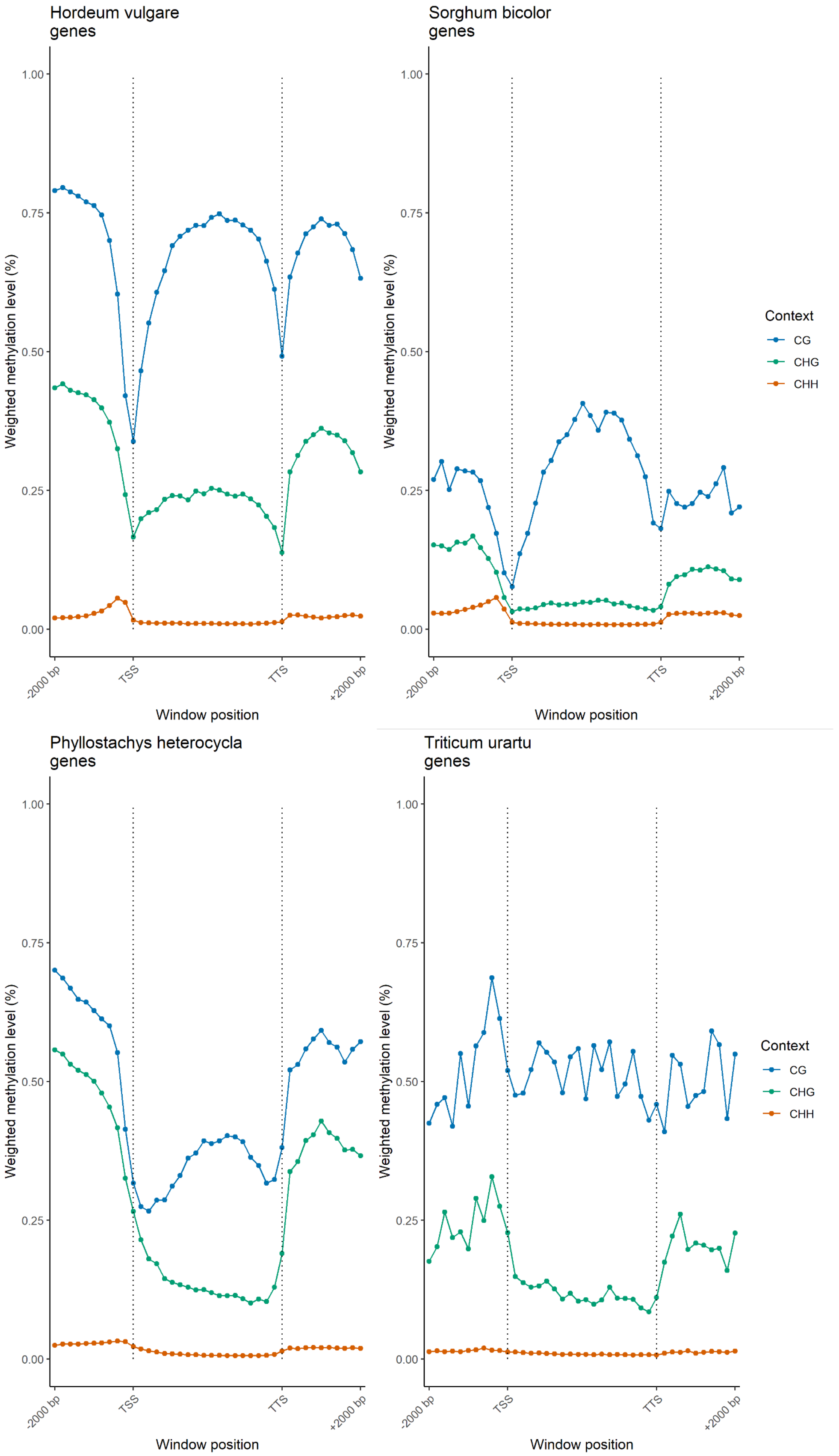

S2. continued

Figure S2. Metaprofiles summarizing methylation levels in all three contexts across genes and near-gene regions. Weighted methylation levels were calculated in 100 bp windows 2 kb upstream and downstream of genes, and transcription start sites (TSS) and transcription termination sites (TTS) are marked by dotted lines.

S3

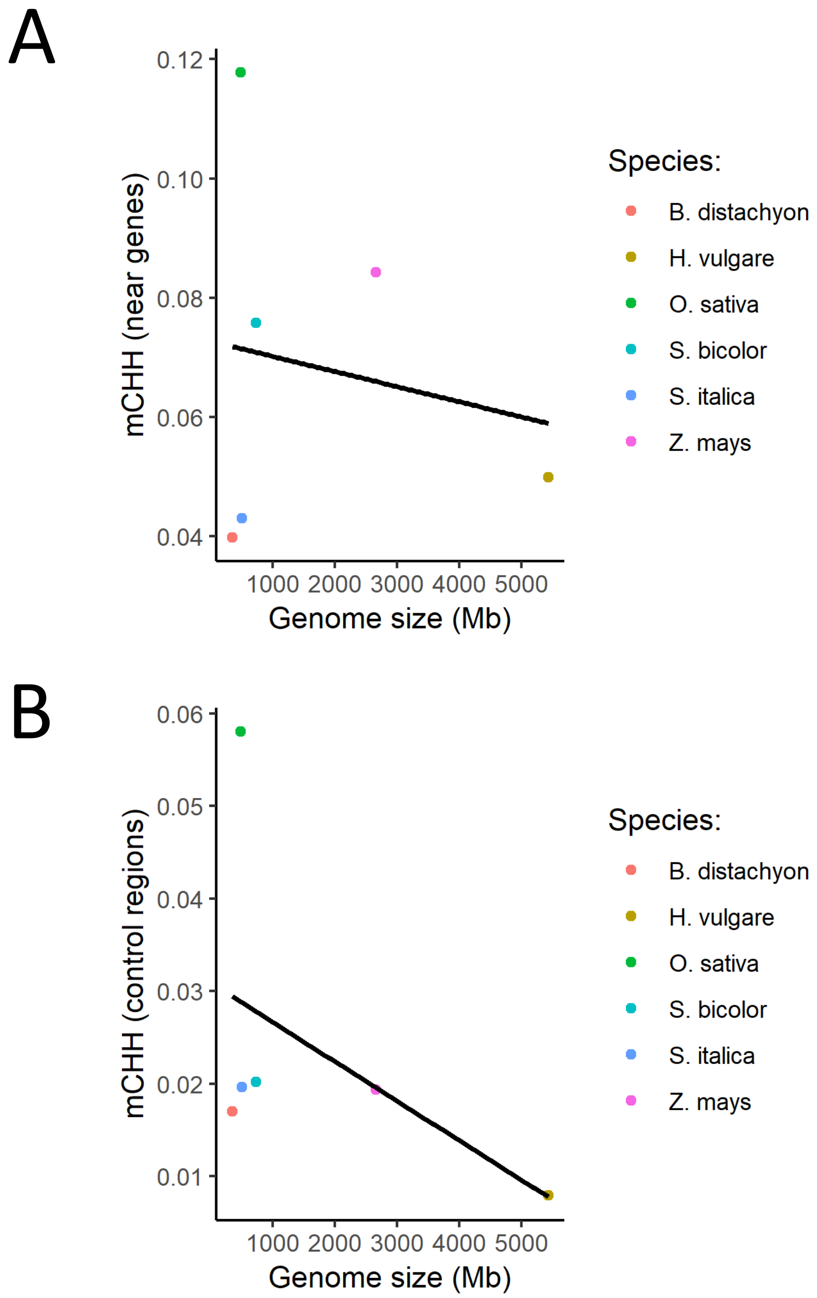

Figure S3. Correlations between GS and mCHH levels near genes (S3a) and among control sites (S3b). These figures represent the numerator and denominator of Figure 1b respectively.

S4

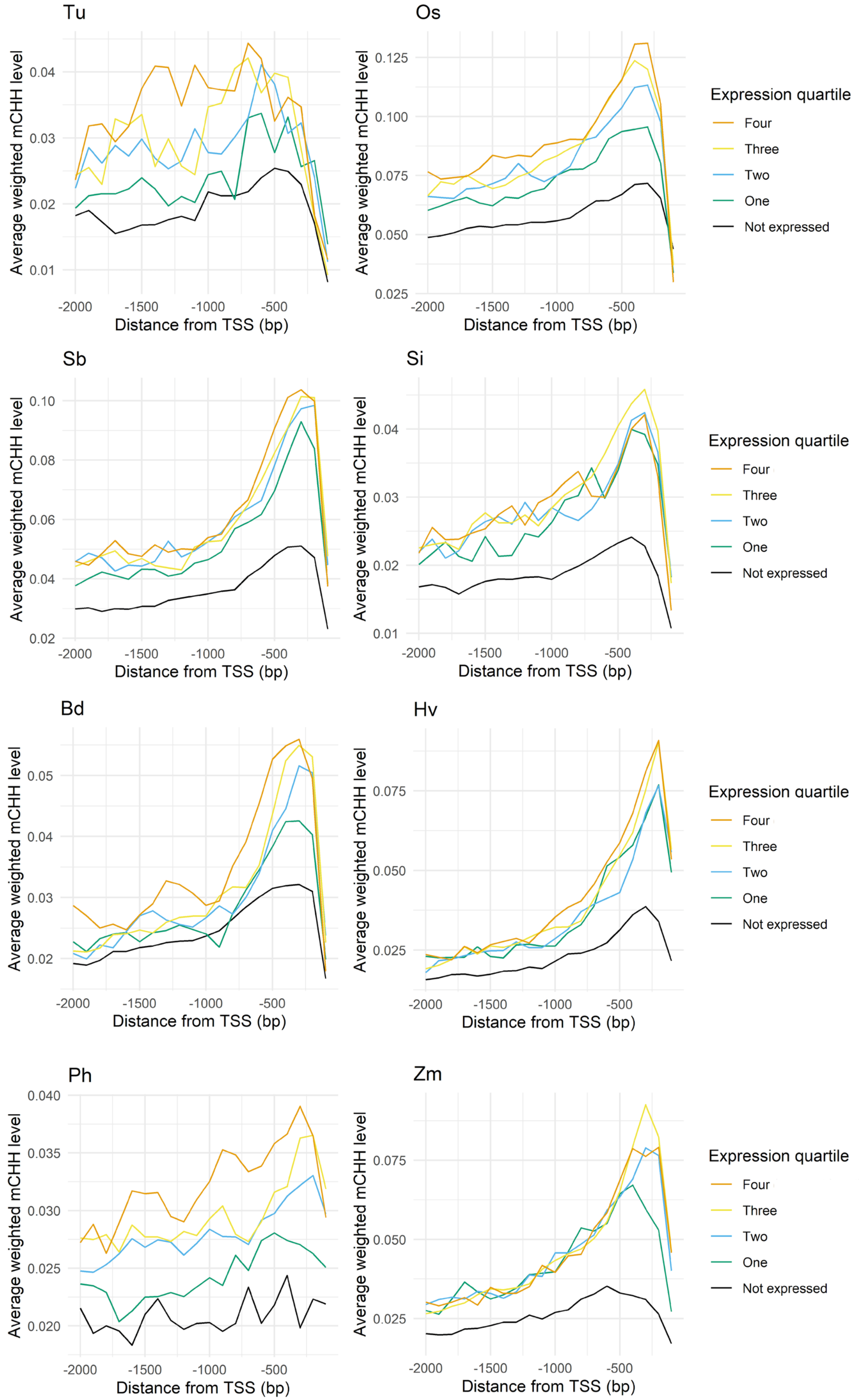

Tu
Os
Sb
Si
Bd
Hv
Ph
Zm
Average weighted mCHH level
Distance from TSS (bp)
-2000
-1500
-1000
-500
Expression quartile
Four
Three
Two
One
Not expressed

Figure S4. Metaprofiles of mCHH levels across 5’ near-gene regions in genes separated into nonexpressed genes and four quartiles of expression (quartile one being those with the lowest expression, and four with highest).

S5

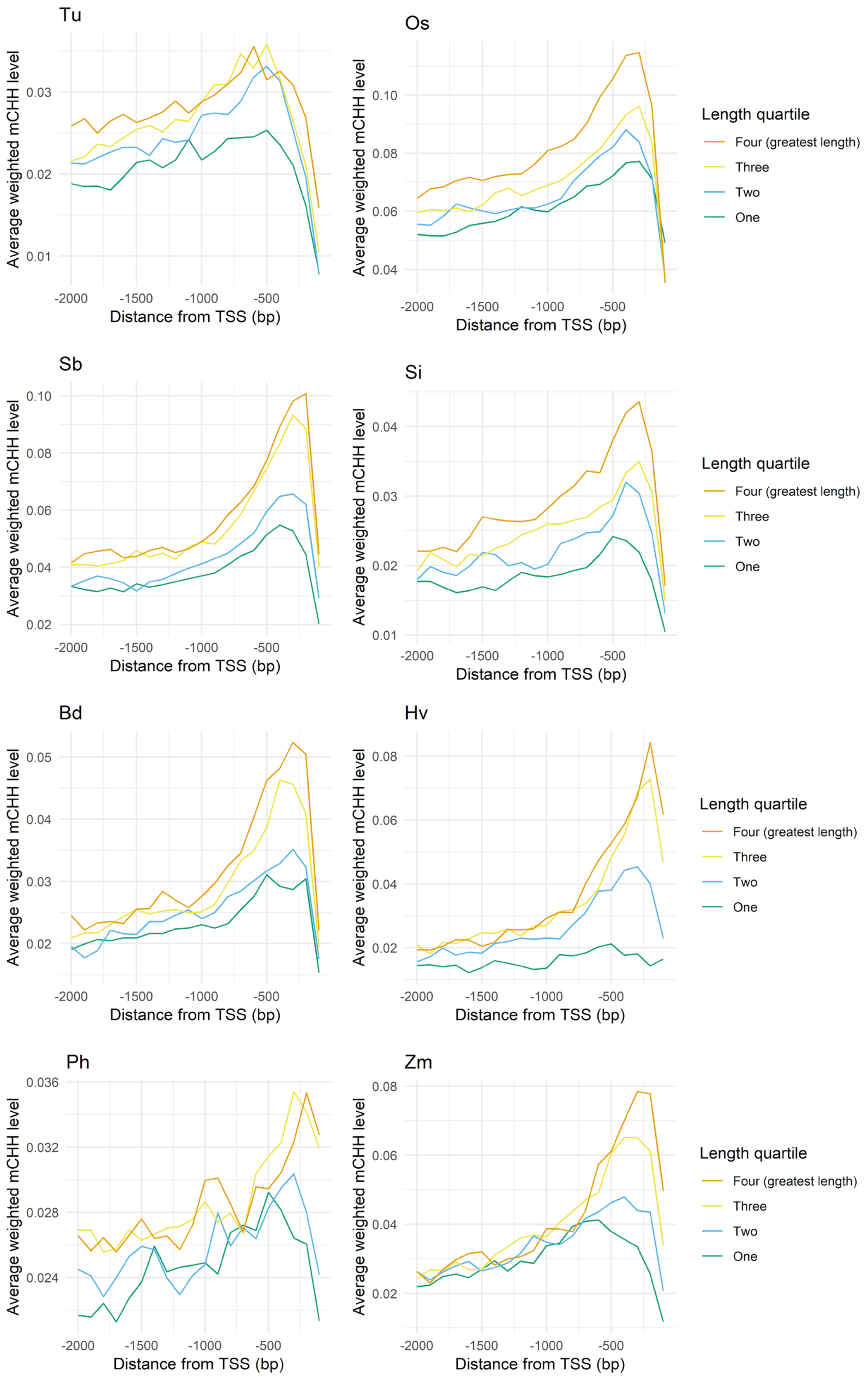

Tu
Os
Sb
Si
Bd
Hv
Ph
Zm
Average weighted mCHH level
Distance from TSS (bp)
-2000
-1500
-1000
-500
Length quartile
Four (greatest length)
Three
Two
One

S5

Figure S5. Metaprofiles of mCHH levels across 5’ near-gene regions in genes separated into four quartiles of length.

S6

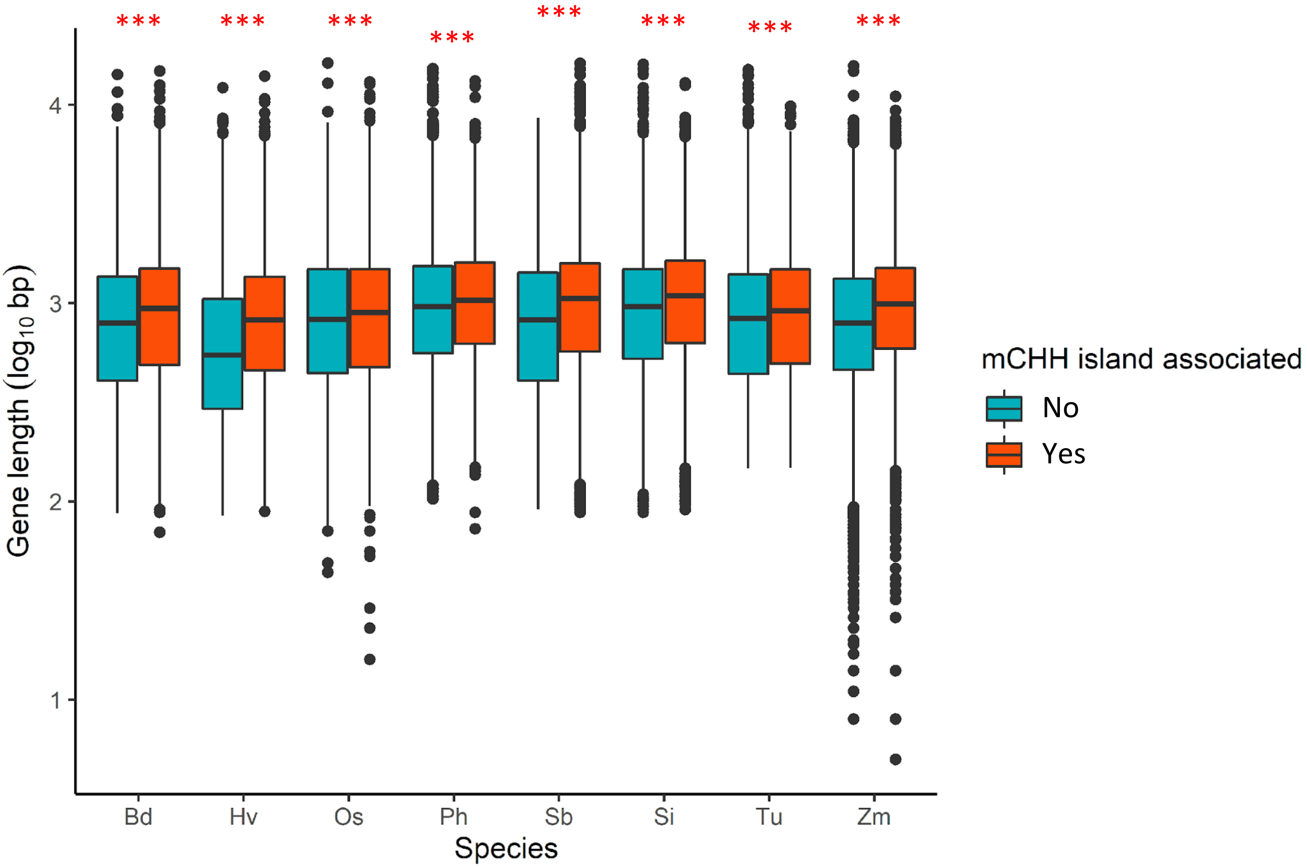

Figure S6. Length of longest transcript between mCHH island associated genes and other genes. Boxplots show the four quartiles of the transcript length distributions in each species, and midde lines represent median. Significance was established by replacing full gene length with transcript length in the logistic regression model from the main text.

S7

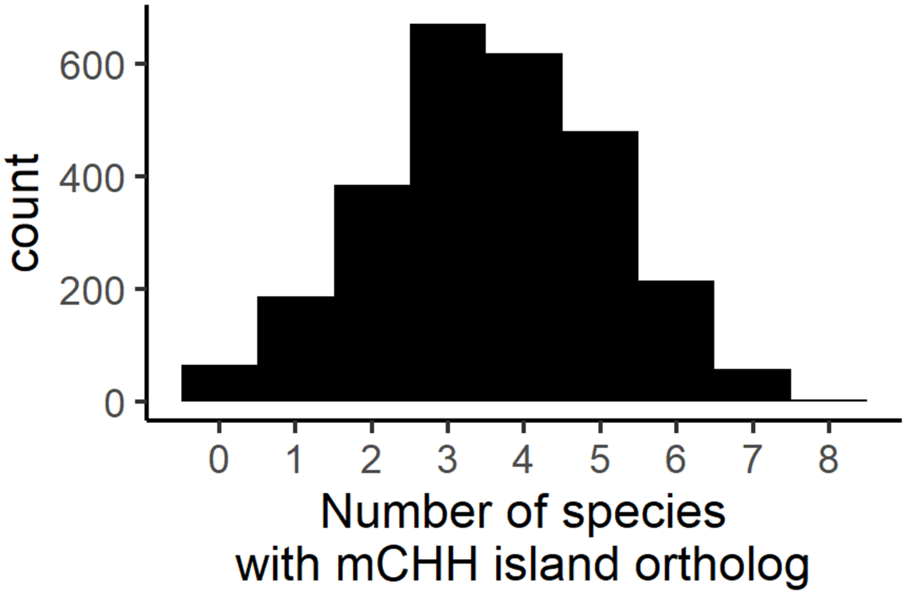

Figure S7. Histogram illustrating the distribution of mCHH island conservation among orthologs of the eight species. The numbers on the x-axis represent the number of species in which orthologs were mCHH island associated. The count on the y-axis is the number of orthologs.

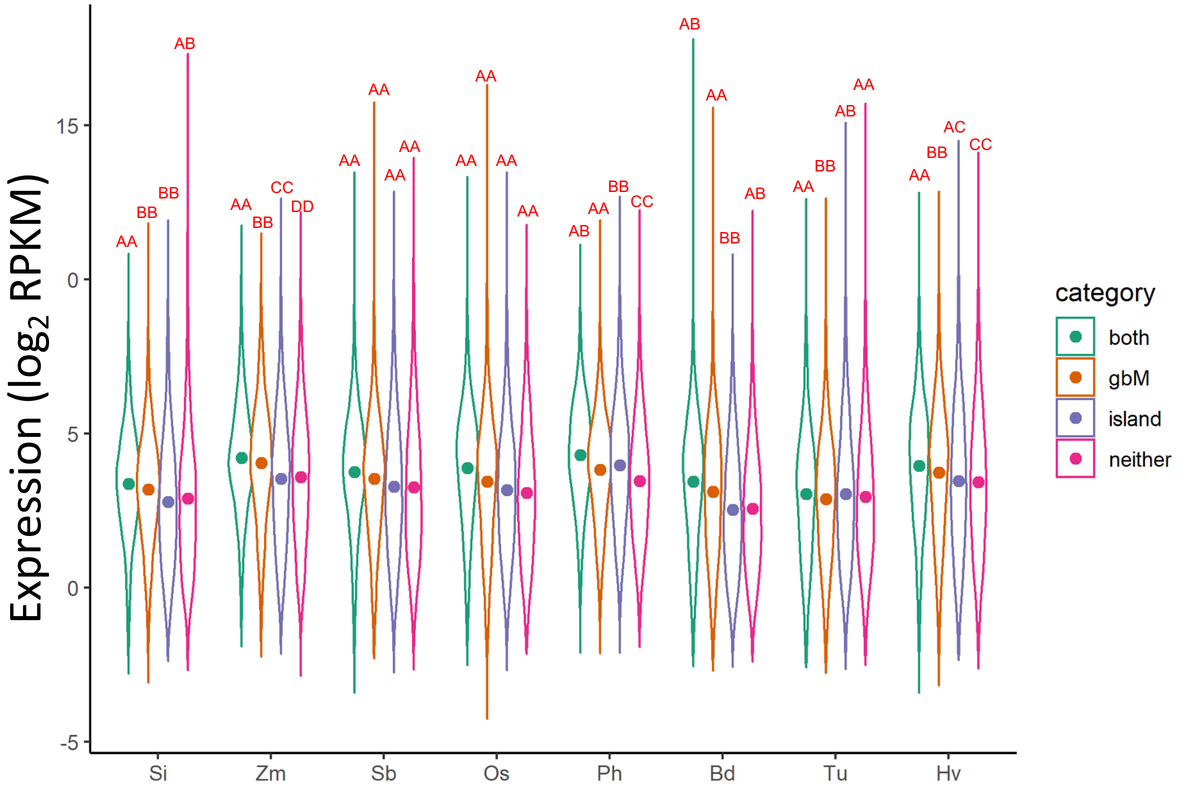

Figure S8. Gene expression levels between categories of gbM genes, mCHH island associated genes, genes with both and genes with neither. Genes were separated into four non-overlapping categories: mCHH island only, gbM only, both island and gbM, and neither. Violin plots depict distributions of expression within each category, with mean values marked by dots. Letter codes represent significance ($P < 0.05$, unpaired t-test), where any distributions that share a letter (e.g., AB and AA) are not significantly different but those that do not share letters (e.g., AA and BB) are significantly different. All comparisons are within, not between, species.

S9

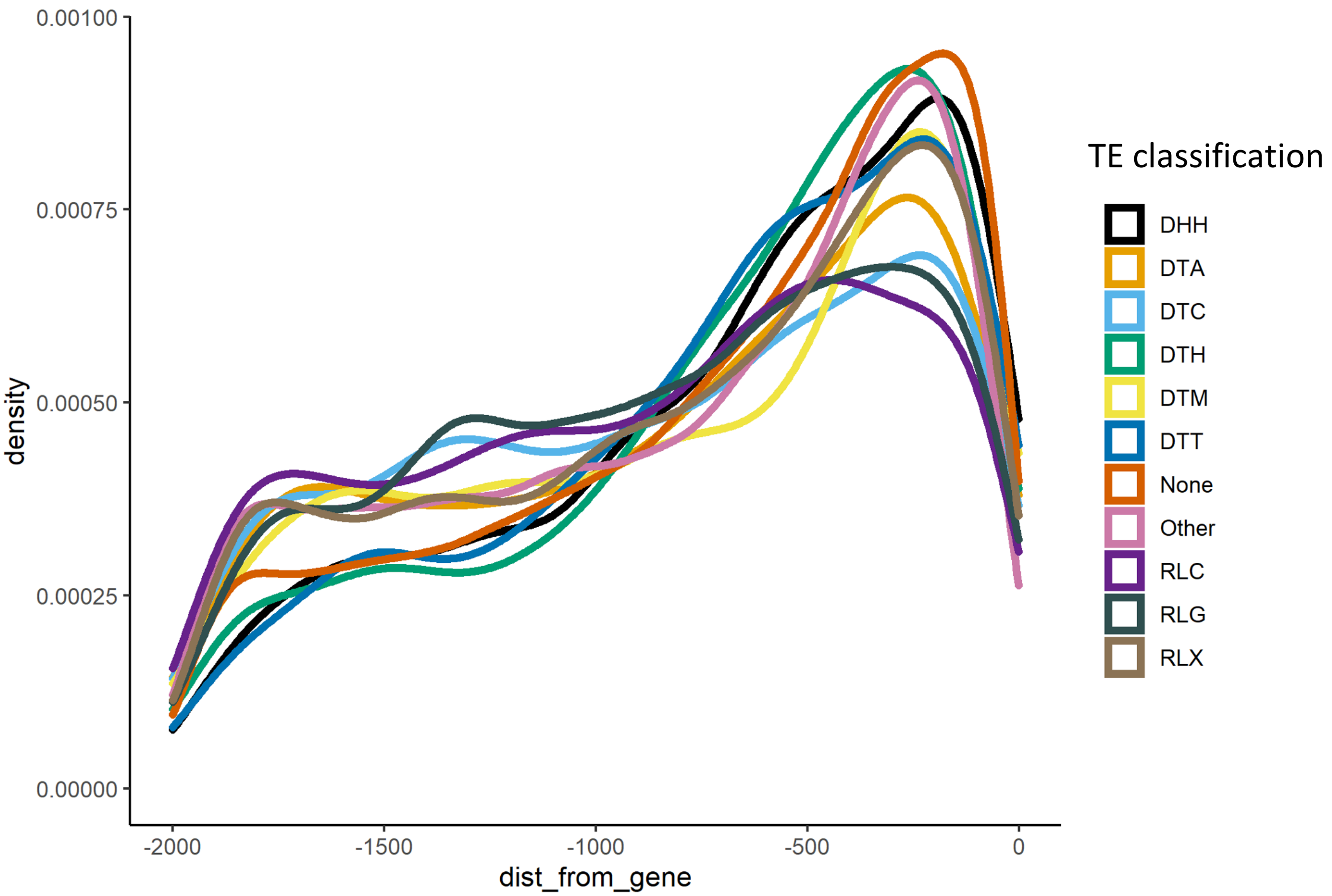

Figure S9. Distributions of distances from the gene TSS to mCHH islands located within different types of TEs and not within TEs ("None"). These distributions include data from all three species.

Supplemental Table 1. Reference statistics

Species	# contigs	Largest contig size (bp)	Total Length (bp)	N50 (bp)	N50 / Total Length
O. sativa	63	4.33E+07	3.75E+08	3.00E+07	0.07988
H. vulgare	10	7.68E+08	4.83E+09	6.57E+08	0.13595
P. heterocycla	277278	4.87E+06	2.05E+09	3.29E+05	0.00016
S. bicolor	867	8.09E+07	7.09E+08	6.87E+07	0.09687
S. italica	336	5.90E+07	4.06E+08	4.73E+07	0.11646
T. urartu	248855	1.07E+06	3.68E+09	8.76E+04	0.00002
Z. mays	267	3.07E+08	2.14E+09	2.24E+08	0.10487
B. distachyon	10	7.51E+07	2.71E+08	5.91E+07	0.21806

Supplemental Table 2. Logistic regression on predictors of mCHH island presence

**Species**	**Expression**		**Distance to nearest TE**		**Gene length**		**Exonic mCG**	
	**Estimate[1]**	***p*-value[2]**	**Estimate**	***p*-value**	**Estimate**	***p*-value**	**Estimate**	***p*-value**
Oryza sativa	-6.43E-01	7.48E-01	-8.54E-04	0.00E+00	7.09E+00	1.13E-111	-1.36E+00	0.00E+00
Sorghum bicolor	-1.81E+00	3.00E-01	-7.42E-04	0.00E+00	5.62E+00	9.31E-61	-7.35E-01	3.57E-86
Zea mays	2.94E+00	3.12E-05	-3.76E-05	2.52E-41	3.38E+00	1.03E-39	-1.60E+00	0.00E+00
Hordeum vulgare	2.62E+00	3.50E-02	-3.19E-04	9.78E-187	8.93E-01	6.57E-02	-1.08E+00	3.59E-257
Brachypodium distachyon	4.59E-01	8.18E-01	-5.37E-05	1.71E-47	3.74E+00	6.98E-81	-6.44E-01	4.58E-83
Setaria italica	-8.05E-01	7.82E-01	-7.64E-04	0.00E+00	3.53E+00	4.61E-47	-3.69E-01	1.32E-17
Triticum urartu	5.85E-01	6.89E-01	-2.19E-04	4.80E-16	1.38E+00	5.65E-04	-8.38E-01	1.45E-101
Phyllostachys heterocycla	1.03E+01	6.77E-21	-4.03E-04	1.43E-68	4.33E-01	6.45E-02	-7.61E-01	2.03E-45

1 Estimate of the effect of the predictor

2 P-value of significance for the estimated effect of the predictor

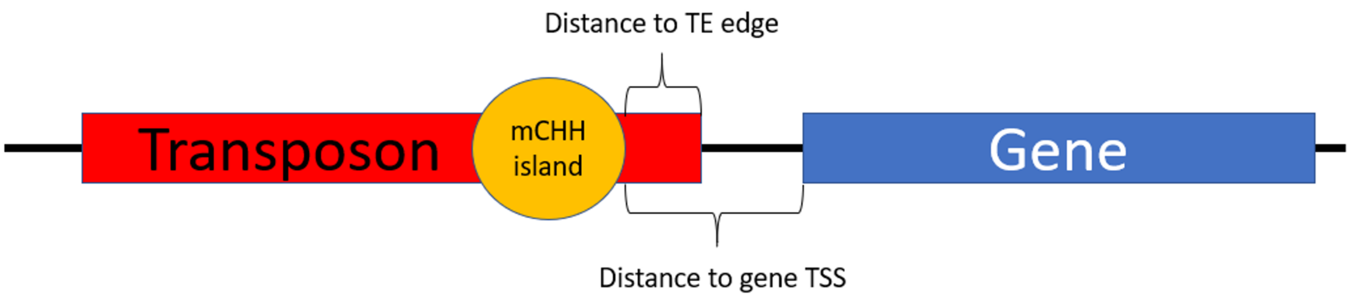

Supplemental Table 3. Coefficients of variation in mCHH island-gene distance vs mCHH island-TE edge distance

Species[1]	TE classification[2]	Gene distance CV	TE distance CV	P value[3]	Test statistic[3]	# Cases[4]	Mean distance to TE edge	Mean distance to gene TSS
Hv	Other	0.80	1.77	0	4.83E+04	2527	81.26	672.78
Hv	DTM	0.86	0.91	0	8.17E+05	119	77.18	660.48
Hv	RLX	0.76	1.23	0	2.02E+05	3149	127.52	744.76
Hv	RLG	0.69	1.33	0	2.29E+04	696	178.93	813.62
Hv	DTA	NA	NA	NA	NA	1	14.00	93.00
Hv	RLC	0.66	1.16	0	4.39E+04	958	188.97	862.32
Hv	DTC	0.74	1.27	0	3.58E+04	732	110.21	776.24
Hv	DTT	0.82	0.77	0	5.53E+05	124	38.51	643.79
Hv	DTH	0.78	1.08	0	3.26E+04	213	122.81	656.64
Hv	DHH	0.54	2.09	2.74E-07	2.64E+01	6	143.33	885.17
Os	Other	0.67	1.19	0	6.59E+05	14574	72.15	841.81
Os	DTM	0.67	1.07	0	4.21E+04	600	118.71	852.06
Os	RLX	0.66	1.62	0	7.64E+04	4963	191.86	859.51
Os	RLG	0.60	1.24	0	2.26E+04	821	383.82	935.37
Os	DTA	0.65	1.16	0	8.90E+03	200	88.42	884.95
Os	RLC	0.62	1.49	0	1.10E+04	648	259.71	926.34
Os	DTC	0.58	1.46	0	5.10E+03	334	311.80	977.07
Os	DTT	0.77	0.55	3.26E-127	5.76E+02	5	66.60	948.60
Os	DTH	0.53	1.84	2.23E-20	8.56E+01	13	188.69	935.85
Os	DHH	0.47	0.68	4.47E-75	3.36E+02	4	105.00	396.25
Zm	Other	0.63	1.06	0	3.70E+03	64	135.41	816.39
Zm	DTM	1.05	2.07	0	1.56E+03	64	121.58	515.03
Zm	RLX	0.70	0.89	0	8.44E+04	302	706.97	767.25
Zm	RLG	0.56	1.29	0	1.16E+04	561	234.79	865.79
Zm	DTA	0.73	0.85	0	1.97E+05	305	117.78	778.33
Zm	RLC	0.54	0.98	0	2.00E+04	471	372.23	1012.97
Zm	DTC	0.50	1.16	3.59E-231	1.05E+03	51	115.24	964.25
Zm	DTT	0.70	1.31	0	1.59E+04	425	94.87	731.58
Zm	DTH	0.73	1.11	0	9.19E+04	1049	79.44	728.49
Zm	DHH	0.76	1.03	0	1.95E+05	1149	552.83	683.98

[1] Hv = *Hordeum vulgare*, Os = *Oryza sativa,* Zm = *Zea mays*

[2] TE classification codes from Wicker 2007

[3] Fultz and Miller asymptotic test for CV equality

[4] Total number of TE-mCHH islands analyzed in each category

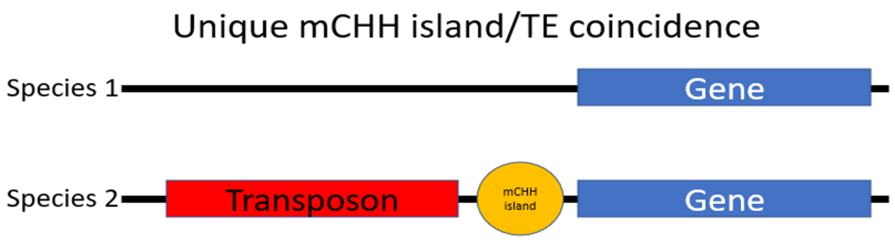

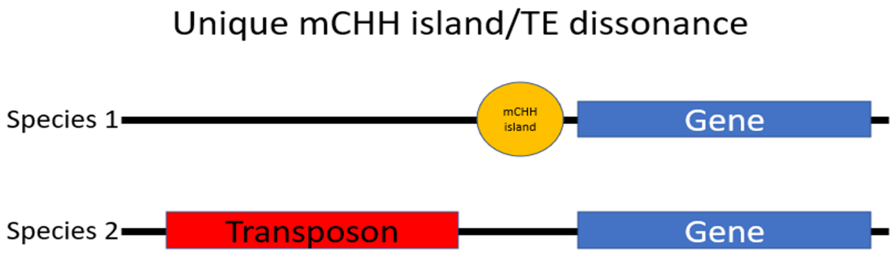

Supplemental Table 4. Coincidence vs dissonance of lineage-specific mCHH islands and TEs

Comparison (TE, species 1, species 2)[1]	Observed unique island/TE coincidence[2]	Expected unique island/TE coincidence[3]	Enrichment unique island/TE coincidence[4]	Observed unique island/TE dissonance	Expected unique island/TE dissonance	Enrichment unique island/TE dissonance	Chi-square statistic[5]	P value[5]
DHH_HvOs	2	2.63	0.76	0	2.25	0.00	7.70	2.609E-01
DHH_ZmHv	87	80.35	1.08	84	81.80	1.03	6.90	3.302E-01
DHH_ZmOs	24	30.92	0.78	166	156.37	1.06	9.56	1.444E-01
DTA_HvOs	10	17.10	0.58	4	4.03	0.99	10.84	9.334E-02
DTA_ZmHv	15	11.79	1.27	7	11.99	0.58	8.97	1.754E-01
DTA_ZmOs	22	20.65	1.07	18	24.81	0.73	21.00	1.265E-02
DTC_HvOs	20	19.43	1.03	80	96.00	0.83	10.48	1.058E-01
DTC_ZmHv	63	52.26	1.21	51	51.40	0.99	14.77	9.746E-02
DTC_ZmOs	7	2.78	2.51	4	5.43	0.74	13.71	3.302E-02
DTH_HvOs	9	5.58	1.61	17	23.14	0.73	9.01	1.731E-01
DTH_ZmHv	91	64.99	1.40	41	65.78	0.62	62.02	5.467E-10
DTH_ZmOs	29	21.00	1.38	69	107.15	0.64	47.43	1.536E-08
DTM_HvOs	39	31.14	1.25	28	32.26	0.87	8.12	2.294E-01
DTM_ZmHv	21	16.51	1.27	16	16.30	0.98	9.46	3.962E-01
DTM_ZmOs	32	27.31	1.17	4	9.93	0.40	18.56	4.970E-03
DTT_HvOs	8	4.20	1.90	14	18.02	0.78	12.34	5.477E-02
DTT_ZmHv	68	60.47	1.12	53	61.26	0.87	11.91	2.187E-01
DTT_ZmOs	22	18.97	1.16	95	100.96	0.94	7.93	5.416E-01
RLC_HvOs	63	71.72	0.88	201	171.07	1.17	19.10	2.432E-02
RLC_ZmHv	119	127.99	0.93	113	127.36	0.89	26.53	1.670E-03
RLC_ZmOs	72	63.94	1.13	110	109.66	1.00	5.57	7.824E-01
RLG_HvOs	56	67.80	0.83	167	147.35	1.13	11.49	2.434E-01
RLG_ZmHv	121	127.79	0.95	133	127.56	1.04	20.18	1.684E-02
RLG_ZmOs	63	67.80	0.93	116	128.26	0.90	8.70	4.650E-01
RLX_HvOs	304	269.22	1.13	335	359.98	0.93	36.97	2.660E-05
RLX_ZmHv	239	216.58	1.10	180	213.27	0.84	17.65	3.948E-02
RLX_ZmOs	308	288.15	1.07	84	93.56	0.90	17.20	4.562E-02

[1] Hv = *Hordeum vulgare*, Os = *Oryza sativa,* Zm = *Zea mays.* TE classification codes from Wicker 2007

[2] See model above for coincidence/dissonance definitions. These numbers represent the observed occurrences in each comparison

[3] Expected values calculated by product of proportions of lineage specific TEs and proportions of lineage specific mCHH islands

[4] Enrichment = observed / expected

[5] Chi-square test of equality between observed and expected proportions of lineage specific mCHH islands and TEs

Supplemental Table 5. Repeat annotations

Species	Repeat annotation	Source
Oryza sativa	irgsp1_repeat_unit.gff	https://rapdb.dna.affrc.go.jp/download/irgsp1.html
Sorghum bicolor	Sbicolor_454_v3.1.1.repeatmasked_assembly_v3.0.1.gff3.gz	https://phytozome.jgi.doe.gov/
Zea mays	B73.structuralTEv2.fulllength.2018-09-19.gff3	https://mcstitzer.github.io/maize_TEs/
Hordeum vulgare	Barley_TE_annotation_v2_18Aug16.tsv	https://doi.org/10.5447/IPK/2016/16
Brachypodium distachyon	Bdistachyon_556_v3.2.repeatmasked_assembly_v3.0.gff3.gz	https://phytozome.jgi.doe.gov/
Setaria italica	Sitalica_312_v2.2.repeatmasked_assembly_v2.gff3.gz	https://phytozome.jgi.doe.gov/
Triticum urartu	Triticum_urartu.GCA_000347455.1.30.gff3	https://plants.ensembl.org/
Phyllostachys heterocycla	P_heterocycla_v1.0.repeats.detail	http://server.ncgr.ac.cn/bamboo/down.php

Supplemental Table 6. Genome information

Species	Bisulfite seq mean coverage	Genome assembly	Source
Oryza sativa	60.3	Osativa_323_v7.0.fa	https://phytozome.jgi.doe.gov/
Sorghum bicolor	17.87	Sbicolor_313_v3.0.fa	https://phytozome.jgi.doe.gov/
Zea mays	6.7	Zea_mays.AGPv4.dna.chromosome.all.fa	http://plants.ensembl.org/index.html
Hordeum vulgare	16.99	150831_barley_pseudomolecules.fa	https://webblast.ipk-gatersleben.de/
Brachypodium distachyon	81.07	Bdistachyon_314_v3.0.fa	https://phytozome.jgi.doe.gov/
Setaria italica	17.88	Sitalica_312_v2.fa	https://phytozome.jgi.doe.gov/
Triticum urartu	14.88	Triticum_urartu.ASM34745v1.31.dna.genome.fa	http://plants.ensembl.org/index.html
Phyllostachys heterocycla	18.17	P_heterocycla_v1.0.Scaffolds.fa	http://server.ncgr.ac.cn/bamboo/
